# Impact of Obesity and Adiposity Measures on Perioperative Outcomes in Gynecologic Malignancy Surgery: A Systematic Review

**DOI:** 10.7759/cureus.114936

**Published:** 2026-08-21

**Authors:** Hassan S Allam, Mohammed Malibary

**Affiliations:** 1 Obstetrics and Gynecology, King Abdulaziz University, Jeddah, SAU; 2 Obstetrics and Gynecology, King Abdulaziz University Hospital, Jeddah, SAU

**Keywords:** body mass index, gynecologic oncology, obesity, perioperative morbidity, postoperative complications, systematic review, visceral obesity

## Abstract

Obesity is associated with increased perioperative morbidity and greater operative complexity in gynecologic oncology surgery. Minimally invasive approaches may reduce some obesity-related complications, while adiposity and body composition measures may provide more accurate perioperative risk stratification than body mass index (BMI) alone. This systematic review evaluated obesity-related predictors of perioperative morbidity following gynecologic oncology surgery, with a specific objective of comparing BMI with alternative adiposity- and body-composition-based measures as predictors of perioperative outcomes. This systematic review was conducted according to the Preferred Reporting Items for Systematic Reviews and Meta-analyses (PRISMA) 2020 guidelines and was registered with the Open Science Framework (OSF) Registry (doi:10.17605/OSF.IO/4TY9B). Several databases were searched for studies evaluating obesity, BMI, and adiposity-related measures as predictors of perioperative morbidity after gynecologic oncology surgery. Records were screened in *Rayyan*, data were extracted using standardized forms, and risk of bias was assessed using Joanna Briggs Institute tools. Among 371 screened records, 73 studies were included in the final analytical synthesis. Endometrial cancer represented the largest evidence group. Obesity, elevated BMI, and adiposity-related measures were most consistently associated with wound complications, surgical site infection, venous thromboembolism, lymphatic morbidity, operative difficulty, and prolonged recovery-related outcomes. These associations were particularly evident during open surgery and high-complexity procedures. Minimally invasive and robotic-assisted approaches generally demonstrated favorable perioperative outcomes in selected obese and morbidly obese patients. Direction-of-evidence mapping qualitatively showed that most included studies reported significant associations between obesity-related measures and perioperative morbidity. CT-defined visceral obesity, waist-to-hip ratio, perinephric fat, and body composition measures frequently provided additional perioperative risk stratification beyond BMI alone. Obesity, elevated BMI, and adiposity-related body composition measures are important predictors of perioperative morbidity in gynecologic oncology surgery. Minimally invasive and robotic approaches may reduce selected obesity-related complications, while CT-derived visceral adiposity and body composition parameters appear to offer more precise perioperative risk stratification than BMI alone, supporting individualized surgical planning and perioperative care. However, these findings are based predominantly on heterogeneous observational studies and should be interpreted cautiously until confirmed by prospective research.

## Introduction and background

Obesity is highly prevalent among patients with gynecologic malignancies and represents an important factor influencing perioperative counseling, surgical planning, anesthetic management, and postoperative recovery [1-3]. In gynecologic oncology surgery, obesity may increase operative complexity through difficult airway management, patient-positioning limitations, increased abdominal wall thickness, restricted surgical exposure, and technical challenges during lymph node assessment and wound closure [3-5]. These difficulties become particularly relevant during extensive procedures such as hysterectomy, pelvic lymphadenectomy, cytoreductive surgery, laparotomy, and robotic-assisted surgery [3,6-9]. Obese patients may additionally experience delayed postoperative mobilization, impaired pulmonary function, and prolonged postoperative recovery, particularly in the presence of obesity-associated comorbidities [4-9].

Several studies have demonstrated that obesity is associated with increased perioperative morbidity after gynecologic cancer surgery [1-5]. Reported complications include wound infection and dehiscence, venous thromboembolism, prolonged operative time, and increased length of hospital stay [1-15]. In endometrial cancer surgery, these concerns are particularly important because obesity frequently coexists with hypertension, diabetes, and cardiovascular disease, making this population especially relevant for studying perioperative risk [16-19]. Consequently, endometrial cancer has become one of the most extensively investigated malignancies regarding obesity-associated surgical morbidity [2,3,16-18].

To reduce perioperative morbidity in obese patients, minimally invasive and robotic-assisted approaches have increasingly been adopted in gynecologic oncology practice [2-4,6-9]. Comparative studies demonstrated that laparoscopic and robotic surgery may reduce blood loss, shorten hospitalization, decrease wound morbidity, and improve postoperative recovery compared with open surgery in obese and morbidly obese patients [2-4,6-9]. Robotic surgery has additionally been shown to remain feasible and relatively safe in severely obese endometrial cancer patients [6-9]. However, increasing obesity may still prolong operative time and increase technical surgical difficulty despite advances in minimally invasive techniques [4,7-9].

Body mass index (BMI) remains the most widely used measure of obesity in clinical practice and surgical research because of its simplicity and accessibility [7]. Nevertheless, BMI alone may inadequately characterize obesity-related perioperative risk [7-9]. Patients with similar BMI values may differ substantially in visceral adiposity, central obesity, fat distribution, and overall body composition [8,9]. These differences may influence anesthetic tolerance, operative exposure, wound healing, and postoperative recovery [8-10]. Therefore, increasing attention has been directed toward alternative adiposity-related measures that may provide additional perioperative risk stratification beyond BMI alone [10-15]. Because BMI cannot distinguish visceral from subcutaneous fat or otherwise capture overall body composition, imaging-based adiposity measures may more directly reflect the physiological burden relevant to perioperative risk; whether these measures meaningfully improve risk prediction beyond BMI in gynecologic oncology surgery represented an important knowledge gap that this review sought to address.

Recent studies have evaluated CT-defined visceral obesity, waist-to-hip ratio, perinephric fat thickness, and CT-based body composition analysis as predictors of perioperative morbidity in gynecologic oncology surgery [10-15]. In endometrial cancer surgery, CT-derived adiposity measurements have been associated with pulmonary intolerance and increased operative difficulty during minimally invasive procedures [10-12]. Similarly, studies evaluating ovarian cancer surgery demonstrated that visceral obesity and adverse body composition profiles may predict postoperative fever, delayed recovery, prolonged hospitalization, and severe postoperative complications more accurately than BMI alone [13-15]. Although obesity-associated perioperative morbidity has been most extensively investigated in endometrial cancer, obesity-related surgical risk is not limited to this malignancy [16-24]. Patients undergoing surgery for cervical, uterine, fallopian tube, primary peritoneal, and mixed gynecologic malignancies may also experience substantial obesity-associated perioperative challenges [24-30]. These risks appear particularly relevant during high-complexity procedures [30-35]. In cervical cancer surgery, elevated BMI has additionally been linked to venous thromboembolism, postoperative infection, and lower-extremity lymphedema [35-45].

Despite increasing evidence, substantial heterogeneity persists regarding cancer type, obesity definition, adiposity assessment, surgical approach, perioperative management, and reported outcomes. Moreover, minimally invasive and robotic-assisted surgery have altered the perioperative impact of obesity in gynecologic oncology. Therefore, this systematic review evaluated obesity, BMI, and adiposity-related measures as predictors of perioperative morbidity after gynecologic oncology surgery.

## Review

Methods

Study Registration and Search Strategy 

This systematic review was registered in Open Science Framework (OSF) Registries on May 16, 2026 (doi:10.17605/OSF.IO/4TY9B). The OSF registration was used to transparently document the review protocol, eligibility criteria, outcomes, and review stage. PROSPERO registration was not pursued because data extraction had already commenced at the time of registration. The review was designed and reported in accordance with the Preferred Reporting Items for Systematic Reviews and Meta-Analyses (PRISMA) 2020 [46]. The PRISMA checklist was followed throughout study design, literature selection, data extraction, and synthesis and is provided as Supplementary Files.

A comprehensive literature search was conducted using PubMed/MEDLINE, Embase, Scopus, Web of Science, and Google Scholar to identify studies published before January 2026 that evaluated obesity, BMI, adiposity-related measures, or body composition parameters as predictors of perioperative morbidity after gynecologic oncology surgery. Data from eligible studies were extracted. The search strategy combined controlled vocabulary and free-text terms related to obesity, adiposity, gynecologic malignancies, surgical procedures, and perioperative outcomes. Search terms included combinations of obesity, obese, overweight, BMI, morbid obesity, class III obesity, visceral obesity, adiposity, body composition, waist-to-hip ratio, perinephric fat, gynecologic cancer, endometrial cancer, ovarian cancer, cervical cancer, vulvar cancer, vaginal cancer, fallopian tube cancer, primary peritoneal cancer, uterine carcinosarcoma, hysterectomy, laparoscopy, laparotomy, robotic surgery, postoperative complications, wound complications, surgical site infection, venous thromboembolism, lymphatic complications, operative time, blood loss, conversion to laparotomy, readmission, and length of hospital stay. Synonyms and related terms were combined using the Boolean operator “OR,” whereas different concepts were intersected using “AND” to optimize search sensitivity and specificity across databases. The complete, database-specific search strings for each of the five databases are provided in full in the Supplementary Files to allow independent replication of the search.

Eligible study designs included randomized trials, prospective and retrospective cohort studies, case-control studies, registry- or database-based analyses, prediction-model studies, economic or cost-effectiveness studies reporting perioperative outcomes, and other observational designs. Case reports, narrative reviews, editorials, letters, protocols, animal studies, laboratory/basic science investigations, and studies lacking obesity-, BMI, or adiposity-related perioperative outcome analyses were excluded.

**Figure 1:**
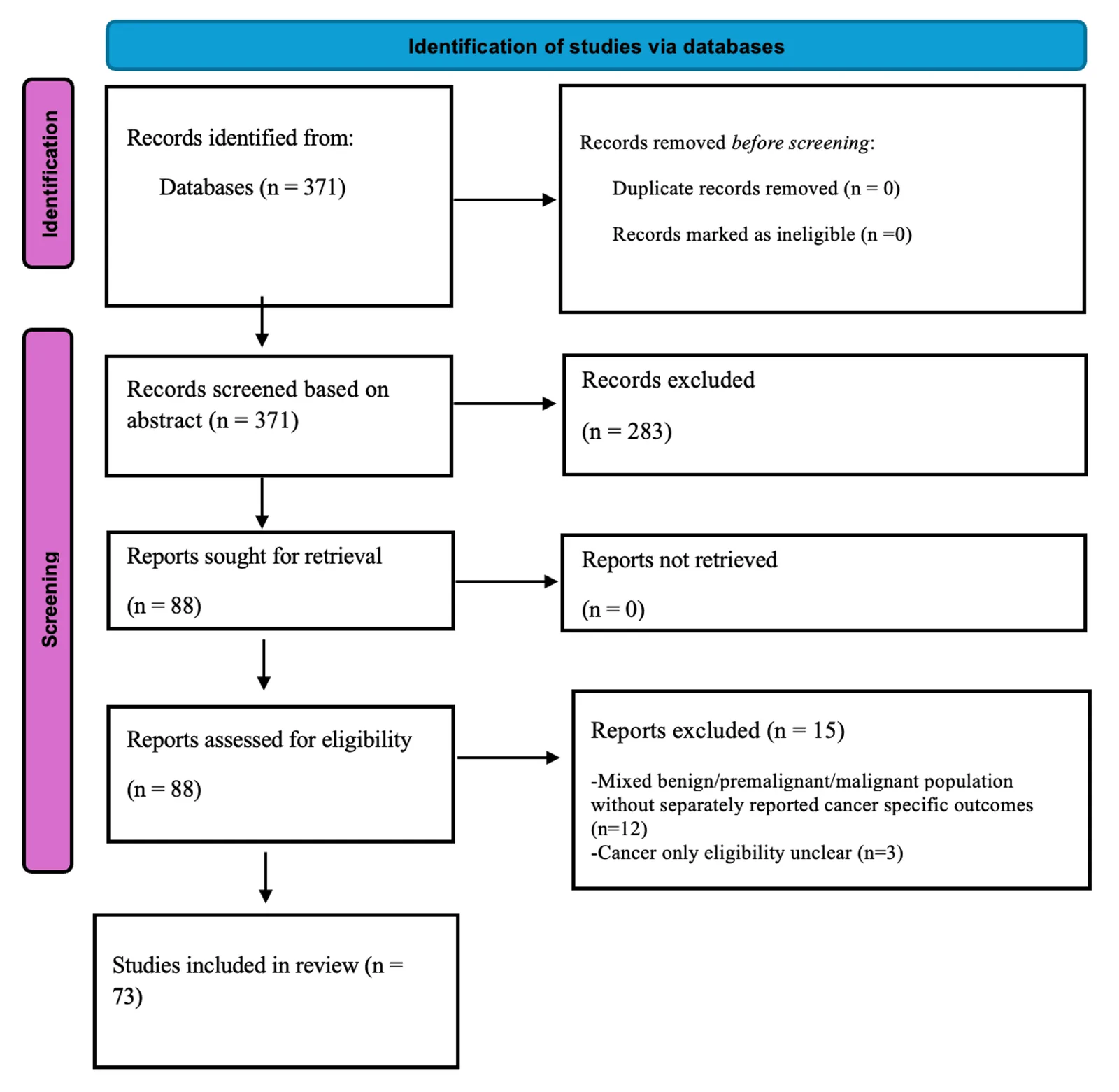
PRISMA flow diagram of study selection PRISMA: Preferred Reporting Items for Systematic Reviews and Meta-analyses

Retrieved records were exported to MS Excel (Microsoft Corporation, Redmond, Washington, United States) and subsequently imported into Rayyan for title and abstract screening. Reference lists of included articles and relevant reviews were additionally screened manually to identify potentially eligible studies not captured through the primary search strategy. Duplicate detection was performed within Rayyan; however, the exact number of duplicate records removed was not displayed by the platform. The study selection process is summarized in the PRISMA flow diagram (Figure 1).

Inclusion and Exclusion Criteria

Studies were selected according to predefined inclusion and exclusion criteria based on the patient, intervention, comparison, outcome, and study design (PICOS) framework [47]. Eligible studies included patients diagnosed with gynecologic malignancies who underwent surgical treatment, surgical staging, or cytoreductive surgery. Eligible malignancies included endometrial, ovarian, cervical, vulvar, vaginal, fallopian tube, and primary peritoneal cancers, uterine carcinosarcoma, and mixed gynecologic malignancy cohorts when cancer-specific outcomes were clearly available. Studies including mixed benign, premalignant, suspected malignant, and malignant populations were eligible only when cancer-specific outcomes were reported separately or when the study population was clearly restricted to malignant disease.

The exposures of interest included obesity, overweight, BMI categories, morbid obesity, visceral obesity, central adiposity, sarcopenic obesity, waist-to-hip ratio, perinephric fat, CT-defined adiposity, CT morphometric parameters, and other adiposity- or body composition-related measures. Comparator groups included non-obese, lower-BMI, or lower-adiposity cohorts whenever available. Eligible outcomes included perioperative and postoperative morbidity measures such as wound complications, surgical site infection, venous thromboembolism, pulmonary complications, urinary complications, lymphatic morbidity, operative difficulty, estimated blood loss, operative duration, conversion to laparotomy, readmission, transfusion, reoperation, length of hospital stay, severe postoperative complications, mortality, and delay to adjuvant therapy.

Studies were excluded if they did not focus on gynecologic malignancy, did not meet the cancer-only population criterion, included mixed benign or premalignant populations without separate cancer-specific outcomes, mentioned obesity or BMI only as a baseline characteristic without outcome analysis, did not report relevant perioperative surgical outcomes, or did not provide sufficient information to confirm eligibility. Studies excluded after eligibility reassessment were documented with reasons for exclusion. 

Data Extraction

Data were extracted using a standardized data extraction spreadsheet. Extracted variables included study number, first author, year of publication, journal, DOI, country, study design, cancer type, surgical procedure or approach, obesity or adiposity definition, comparator group, sample size, outcomes reported, effect estimates or key numerical findings when available, main findings, full-text availability, inclusion status, synthesis group, and reason for exclusion where applicable. When studies reported multiple outcomes, perioperative morbidity outcomes most relevant to obesity, BMI, or adiposity were prioritized for synthesis. Studies with potentially overlapping cohorts were interpreted cautiously to avoid double-counting in the narrative synthesis.

Potentially overlapping cohorts were identified by cross-referencing study institution or research group, recruitment period, sample size, and registry or trial name (e.g., the RISC-GYN cohort and GOG/NRG Oncology datasets); where overlap was suspected, the corresponding findings were flagged and interpreted cautiously in the narrative synthesis rather than being treated as independent evidence.

Risk-of-Bias Assessment

Risk of bias was assessed for included studies using the Joanna Briggs Institute (JBI) critical appraisal tools according to study design [48]. Cohort studies, case-control studies, cross-sectional studies, randomized trials, and case series were assessed using the appropriate JBI checklist. Studies excluded after eligibility reassessment were not included in the risk-of-bias synthesis. One included economic/cost-effectiveness study was not assessed using JBI because it was not a clinical observational outcomes study. Overall risk of bias was categorized as low, moderate, or high based on the number of criteria adequately fulfilled and the extent of concerns related to selection bias, exposure measurement, outcome ascertainment, confounder adjustment, follow-up, and reporting completeness. 

Data Analysis

Data synthesis was performed using a structured analytical narrative approach because substantial clinical and methodological heterogeneity was anticipated across included studies. Heterogeneity involved cancer type, surgical procedure, obesity definition, adiposity assessment, perioperative management, outcome definitions, and reported effect estimates. Findings were therefore summarized according to cancer type, surgical approach, adiposity-related exposure, and perioperative morbidity category. When studies reported comparable outcomes and effect measures, potential subgroup or quantitative synthesis was considered. However, because of variability in exposure definitions, statistical adjustment, and outcome reporting, the primary synthesis remained narrative rather than meta-analytic. Studies were additionally categorized according to the direction and significance of reported associations. 

The specific sources of heterogeneity precluding quantitative pooling included differences in how obesity and adiposity were defined and categorized (e.g., BMI thresholds versus imaging-derived cutoffs), variation in comparator groups, differing perioperative morbidity definitions and follow-up windows, and inconsistent statistical adjustment for confounders across study designs. For the qualitative direction-of-evidence mapping (Figure 4), each study was categorized by two reviewers as showing a significant association, a positive narrative trend without formal statistical testing, no significant association, or unclear/nontestable evidence, based on the direction and statistical reporting of its obesity/BMI/adiposity-outcome relationship; disagreements were resolved by discussion. This classification summarized the direction of reported findings rather than their magnitude or study quality, and studies were not weighted by sample size, precision, or risk-of-bias rating, which is an inherent limitation of vote-counting approaches to evidence synthesis.

Results

A total of 371 records were screened in Rayyan following duplicate detection and removal. During title and abstract screening, 283 records were excluded. Eighty-eight reports were retrieved and assessed for eligibility at the full-text or abstract level according to data availability, with no unretrieved reports. After detailed eligibility reassessment, 15 reports were excluded, resulting in 73 studies included in the final systematic review and analytical synthesis, as illustrated in PRISMA (Figure 1). The main reasons for exclusion were summarized in the Supplementary Table S2 in the Appendix. Excluded studies primarily involved mixed benign, premalignant, or noncancer populations without separately reported gynecologic malignancy-specific outcomes; unclear cancer-only eligibility; duplicated or potentially overlapping datasets; or primary outcomes not directly related to perioperative morbidity.

Study Characteristics

**Figure 2:**
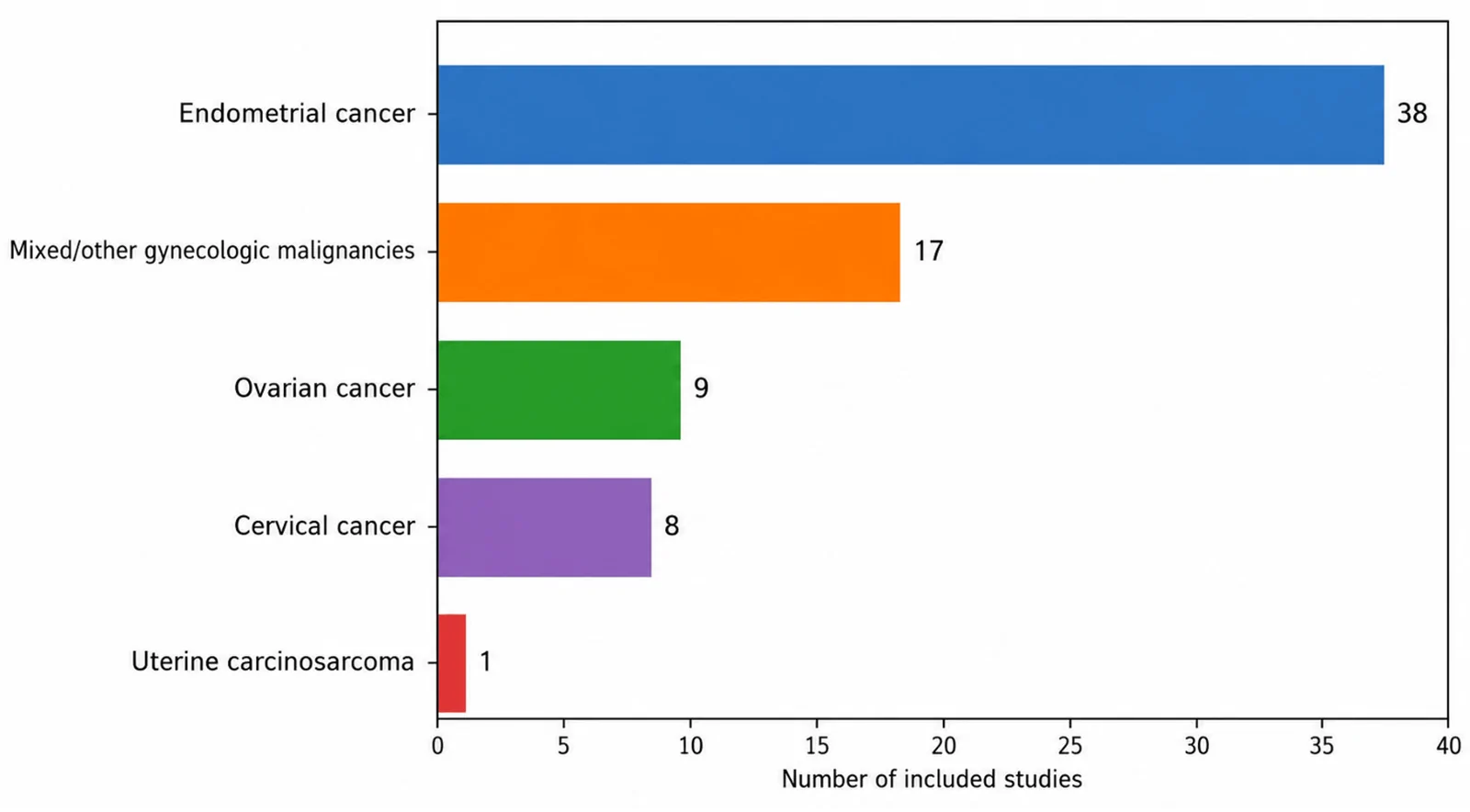
Included studies by cancer groups

**Figure 3:**
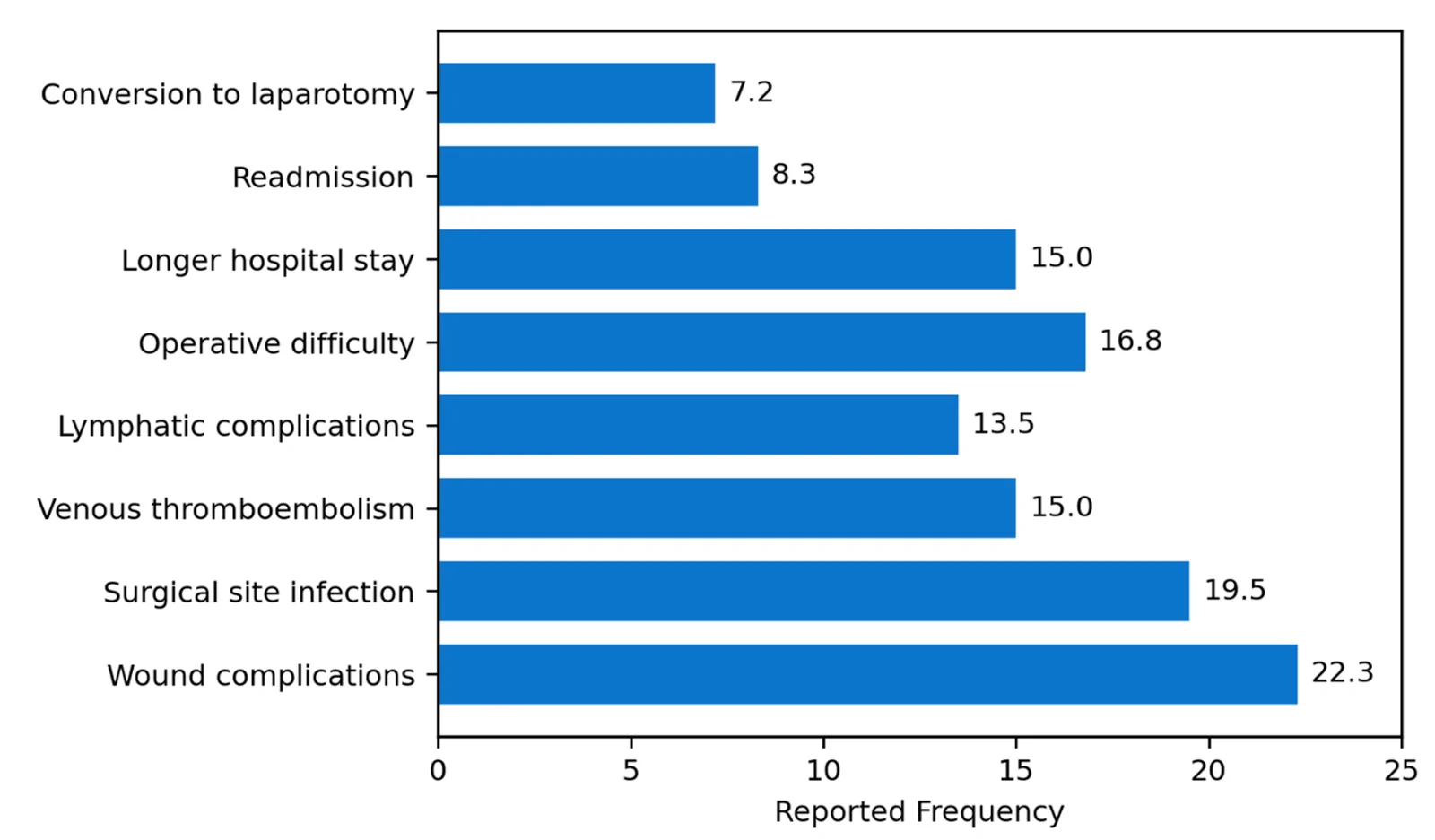
Most common obesity-associated perioperative morbidities

The characteristics of the included studies were summarized in the Supplementary Table S1 in the Appendix. The 73 included studies were published between 2014 and 2026 and predominantly consisted of observational evidence. Endometrial cancer represented the largest evidence group, followed by ovarian cancer, cervical cancer, mixed gynecologic malignancy cohorts, uterine carcinosarcoma, fallopian tube cancer, and primary peritoneal cancer (Figure 2). The distribution of commonly reported obesity-associated perioperative morbidities was summarized in Figure 3. 

Risk of Bias

The risk-of-bias assessment was summarized in Table 1. Risk of bias was assessed for 72 of the 73 included studies using JBI critical appraisal tools according to study design. One economic/cost-effectiveness study was not evaluated using JBI because it did not represent a clinical observational outcomes study. Overall, 63 studies (86.3%) were judged to have low risk of bias, 10 studies (13.7%) had moderate risk of bias, and no included study was categorized as having high risk of bias. Moderate-risk studies were most commonly limited by incomplete adjustment for confounding variables, limited follow-up duration, or incomplete perioperative outcome ascertainment. Studies excluded after eligibility reassessment were not included in the risk-of-bias synthesis.

**Table 1:** Risk-of-bias assessment of the included studies using the JBI critical appraisal tools JBI: Joanna Briggs Institute Y = yes (criterion met); U = unclear; N = no; — = not applicable. JBI critical appraisal domains: Q1, groups similar/same population; Q2, exposure measured reliably; Q3, exposure clearly defined; Q4, confounders identified; Q5, confounders addressed; Q6, outcomes measured reliably; Q7, follow-up adequate; Q8, appropriate statistical analysis. Overall risk: low = mostly yes; moderate = some important unclear/no; high = many important concerns. Of the 73 included studies appraised, 63 were rated low, 10 were moderate, and 0 were high risk

No.	Study (author, year)	JBI tool	Q1	Q2	Q3	Q4	Q5	Q6	Q7	Q8	Overall risk
1	Bogani et al., 2021 [49]	Cohort	Y	Y	Y	Y	Y	Y	U	Y	Low
2	Nhokaew et al., 2015 [1]	Cohort	Y	Y	Y	Y	Y	Y	Y	Y	Low
3	Lechartier et al., 2023 [2]	Cohort	Y	Y	Y	Y	Y	Y	U	Y	Low
4	Liu et al., 2023 [50]	Case-control	Y	Y	Y	U	U	Y	—	Y	Moderate
5	King et al., 2022 [3]	Cohort	Y	Y	Y	Y	Y	Y	U	Y	Low
6	Gambacorti-Passerini et al., 2019 [51]	Cohort	Y	Y	Y	U	U	Y	U	Y	Moderate
7	Pyrzak et al., 2020 [31]	Cohort	Y	Y	Y	Y	Y	Y	Y	Y	Low
8	Abdelbadee et al., 2016 [25]	Cohort	Y	Y	Y	Y	Y	Y	U	Y	Low
9	Wenzel et al., 2020 [52]	Cohort	Y	Y	Y	Y	Y	Y	Y	Y	Low
10	Bohn et al., 2022 [53]	A cost-effectiveness analysis	Y	Y	Y	U	U	Y	U	Y	Moderate
11	Durdağ et al., 2023 [4]	Cohort	Y	Y	Y	Y	Y	Y	Y	Y	Low
12	Kılıç et al., 2021 [32]	Cohort	Y	Y	Y	Y	Y	Y	Y	Y	Low
13	Levin et al., 2024 [5]	Cohort	Y	Y	Y	Y	Y	Y	Y	Y	Low
14	Cheng et al., 2024 [33]	Cohort	Y	Y	Y	Y	Y	Y	Y	Y	Low
15	Peng et al., NR [54]	Cohort	Y	Y	Y	Y	Y	Y	Y	Y	Low
16	Lei et al., 2017 [6]	Cohort	Y	Y	Y	Y	Y	Y	Y	Y	Low
17	Milman et al., 2024 [55]	Cohort	Y	Y	Y	Y	Y	Y	Y	Y	Low
18	Palaiologos et al., 2025 [56]	Cohort	Y	Y	Y	U	U	Y	U	Y	Moderate
19	Uccella et al., 2015 [57]	Case-control	Y	Y	Y	Y	Y	Y	U	Y	Low
20	Cybulska et al., 2017 [58]	Cohort	Y	Y	Y	U	U	Y	U	Y	Moderate
21	Januszek et al., 2019 [59]	Cohort	Y	Y	Y	Y	Y	Y	Y	Y	Low
22	Boitano et al., 2021 [34]	Cohort	Y	Y	Y	Y	Y	Y	Y	Y	Low
23	Straubhar et al., 2024 [35]	Cohort	Y	Y	Y	Y	Y	Y	Y	Y	Low
24	Sofer et al., 2020 [7]	Cohort	Y	Y	Y	Y	Y	Y	Y	Y	Low
25	Deura et al., 2019 [8]	Cohort	Y	Y	Y	Y	Y	Y	Y	Y	Low
26	Leitao et al., 2019 [60]	Cohort	Y	Y	Y	Y	Y	Y	U	Y	Low
27	Mendivil et al., 2015 [9]	Cohort	Y	Y	Y	Y	Y	Y	Y	Y	Low
28	Chan et al., 2015 [10]	Cohort	Y	Y	Y	Y	Y	Y	Y	Y	Low
29	Carbajal-Mamani et al., 2021 [61]	Cohort	Y	Y	Y	Y	Y	Y	Y	Y	Low
30	Ayres et al., 2025 [62]	Cohort	Y	Y	Y	U	U	Y	Y	Y	Moderate
31	Salman et al., 2025 [26]	Cohort	Y	Y	Y	Y	Y	Y	U	Y	Low
32	Ozdemir et al., 2022 [63]	Cohort	Y	Y	Y	Y	Y	Y	Y	Y	Low
33	Bebia et al., 2021 [64]	Cohort	Y	Y	Y	Y	Y	Y	Y	Y	Low
34	Gracia et al., 2020 [11]	Cohort	Y	Y	Y	Y	Y	Y	U	Y	Low
35	Ramzan et al., 2015 [65]	Cohort	Y	Y	Y	Y	Y	Y	Y	Y	Low
36	Iavazzo et al., 2021 [66]	Case series	Y	Y	Y	U	—	Y	U	Y	Moderate
37	Kadoch et al., 2024 [12]	Cohort	Y	Y	Y	Y	Y	Y	Y	Y	Low
38	Mendivil et al., 2016 [36]	Cohort	Y	Y	Y	Y	Y	Y	U	Y	Low
39	Mizuno et al., 2025 [13]	Cohort	Y	Y	Y	Y	Y	Y	Y	Y	Low
40	Åkesson et al., 2021 [37]	Cohort	Y	Y	Y	Y	Y	Y	Y	Y	Low
41	Pace et al., 2026 [27]	Cohort	Y	Y	Y	Y	Y	Y	Y	Y	Low
42	Cheng et al., 2016 [14]	Cohort	Y	Y	Y	Y	Y	Y	Y	Y	Low
43	Li et al., 2026 [67]	Analytical cross-sectional	Y	Y	Y	Y	Y	Y	U	Y	Low
44	Cunningham et al., 2015 [15]	Cohort	Y	Y	Y	Y	Y	Y	U	Y	Low
45	Mahdi et al., 2014 [38]	Cohort	Y	Y	Y	Y	Y	Y	Y	Y	Low
46	Nie et al., 2024 [28]	Cohort	Y	Y	Y	Y	Y	Y	U	Y	Low
47	Urunsak et al., 2020 [16]	Cohort	Y	Y	Y	Y	Y	Y	Y	Y	Low
48	Díaz-Feijoo et al., 2020 [68]	RCT	Y	Y	Y	Y	Y	Y	Y	Y	Low
49	Asanoma et al., 2022 [17]	Cohort	Y	Y	Y	Y	Y	Y	U	Y	Low
50	Mahdi et al., 2016 [69]	Cohort	Y	Y	Y	Y	Y	Y	Y	Y	Low
51	Chen et al., 2024 [18]	Cohort	Y	Y	Y	Y	Y	Y	Y	Y	Low
52	Reyes Claret et al., 2020 [70]	Cohort	Y	Y	Y	U	U	Y	U	Y	Moderate
53	Raventós-Tato et al., 2019 [19]	Cohort	Y	Y	Y	Y	Y	Y	U	Y	Low
54	Backes et al., 2015 [20]	Cohort	Y	Y	Y	Y	Y	Y	Y	Y	Low
55	Kim et al., 2022 [39]	Cohort	Y	Y	Y	Y	Y	Y	U	Y	Low
56	Matanes et al., 2021 [71]	Cohort	Y	Y	Y	Y	Y	Y	U	Y	Low
57	Inci et al., 2020 [72]	Cohort	Y	Y	Y	Y	Y	Y	Y	Y	Low
58	Kawai et al., 2021 [21]	Cohort	Y	Y	Y	Y	Y	Y	U	Y	Low
59	Kumar et al., 2015 [40]	Cohort	Y	Y	Y	Y	Y	Y	U	Y	Low
60	Inci et al., 2021 [73]	Cohort	Y	Y	Y	Y	Y	Y	Y	Y	Low
61	Xing et al., 2024 [41]	Cohort	Y	Y	Y	Y	Y	Y	Y	Y	Low
62	de Jong et al., 2024 [42]	Cohort	Y	Y	Y	Y	Y	Y	Y	Y	Low
63	Joshi et al., 2024 [22]	Cohort	Y	Y	Y	Y	Y	Y	Y	Y	Low
64	Simpson et al., 2020 [23]	Cohort	Y	Y	Y	Y	Y	Y	Y	Y	Low
65	Heus et al., 2021 [29]	Cohort	Y	Y	Y	Y	Y	Y	Y	Y	Low
66	Bacalbasa et al., 2020 [74]	Cohort	Y	Y	Y	U	U	Y	Y	Y	Moderate
67	Castro et al., 2018 [43]	Cohort	Y	Y	Y	Y	Y	Y	Y	Y	Low
68	Güner et al., 2024 [75]	Cohort	Y	Y	Y	U	U	Y	U	Y	Moderate
69	Hayes et al., 2017 [44]	Cohort	Y	Y	Y	Y	Y	Y	Y	Y	Low
70	Duska et al., 2015 [45]	Cohort	Y	Y	Y	Y	Y	Y	U	Y	Low
71	Su et al., 2025 [30]	Cohort	Y	Y	Y	Y	Y	Y	Y	Y	Low
72	Xu et al., 2025 [24]	Cohort	Y	Y	Y	Y	Y	Y	U	Y	Low
73	Matsuo et al., 2017 [76]	Cohort	Y	Y	Y	Y	Y	Y	Y	Y	Low

Narrative Synthesis by Outcome and Cancer Type

Endometrial cancer**:** Endometrial cancer represented the largest subgroup among the included studies [2-4,7-17,19-23,63,76,77]. Across studies, minimally invasive and robotic-assisted surgical approaches were generally associated with favorable perioperative outcomes in obese and morbidly obese patients. Several studies demonstrated lower conversion rates, shorter hospital stay, reduced blood loss, and acceptable perioperative complication profiles among obese patients undergoing robotic or laparoscopic surgery compared with open surgery. In contrast, the adverse impact of obesity appeared more clinically significant in laparotomy cohorts, particularly regarding wound morbidity and recovery-related complications, whereas minimally invasive approaches often attenuated obesity-associated perioperative risk [2-4,7-17,19-23,63,77].

Wound complications and surgical site infection were among the most frequently reported obesity-associated outcomes and represented the most common morbidity category overall. Studies evaluating open surgical cohorts consistently demonstrated that higher BMI or morbid obesity increased the risk of wound infection, wound dehiscence, and surgical site complications. Conversely, minimally invasive and robotic approaches were associated with lower wound morbidity, supporting their use when technically feasible [1,4,7,8,16,23,37,38,65].

Several studies specifically assessed robotic surgery in obese and morbidly obese endometrial cancer populations and generally demonstrated that robotic surgery remained feasible and safe across BMI categories, with acceptable rates of lymph node assessment, perioperative complications, and conversion. Although increasing BMI was associated with longer operative time in some reports, this did not consistently translate into higher rates of major postoperative morbidity [2,3,9-13,15,17,19-22]. 

Lymphatic outcomes and nodal assessment were also evaluated in selected cohorts. Obesity and CT-based adiposity measures were associated with lower sentinel lymph node mapping success and higher rates of failed mapping in some studies. Sentinel lymph node strategies appeared to reduce blood loss and operative duration compared with complete lymphadenectomy in obese patients, although findings remained heterogeneous across studies [26,60,71].

Studies using CT-derived body composition and anthropometric adiposity measures suggested that visceral obesity, waist-to-hip ratio, perinephric fat, and other morphometric variables may provide additional perioperative risk stratification beyond BMI alone. These measures were associated with pulmonary intolerance, postoperative complications, and surgical complexity in selected cohorts, supporting the concept that BMI alone may inadequately characterize perioperative risk in endometrial cancer surgery [25-27]. Minimally invasive and robotic techniques appear feasible in obese and morbidly obese patients and may reduce obesity-associated perioperative morbidity compared with laparotomy [1-4,7-17,19-23,37,38,63].

Ovarian, fallopian tube, and primary peritoneal cancer: Studies involving ovarian, fallopian tube, and primary peritoneal cancer primarily evaluated obesity, BMI, visceral adiposity, and CT-defined body composition in relation to cytoreductive surgery, interval or primary debulking surgery, thromboembolic events, postoperative complications, readmission, and recovery-related outcomes [28-30,39-41,43,45,69]. Several studies suggested that obesity and elevated BMI were associated with increased perioperative morbidity in ovarian cancer surgery, particularly in advanced-stage disease requiring extensive cytoreductive procedures. Higher BMI was associated with severe postoperative complications, thrombotic events, readmission, delayed recovery, and early postoperative mortality in selected cohorts. However, the strength and direction of association varied across studies, likely reflecting differences in disease burden, surgical complexity, residual disease, nutritional status, and perioperative management [39-41,43,45,69]. 

CT-based adiposity and body composition measures were repeatedly evaluated in ovarian cancer cohorts. Visceral obesity was associated with postoperative fever, prolonged hospitalization, delayed recovery, and increased complication rates. Several studies suggested that visceral adiposity may outperform BMI alone in predicting postoperative morbidity after extensive cytoreductive surgery [28-30]. 

In advanced ovarian cancer, perioperative outcomes appeared to be influenced by multiple interacting variables, including age, albumin level, nutritional status, operative duration, blood loss, disease stage, surgical complexity, and adiposity measures. Prediction-model studies frequently incorporated BMI into multifactorial risk models for severe complications and early mortality [30,40,41,43]. Venous thromboembolism and readmission outcomes were also reported. Higher BMI was identified as an independent predictor of thrombotic complications and increased readmission risk in selected ovarian cancer cohorts, reinforcing the importance of individualized perioperative risk assessment in obese patients undergoing cytoreductive surgery [39,45]. CT-defined visceral obesity and body composition measures may provide additional perioperative risk information beyond BMI alone [28-30,39-41,43,45,69]. Because two of the included RISC-GYN studies may represent overlapping datasets, their findings were interpreted cautiously during narrative synthesis to avoid potential double counting.

Cervical cancer:** **Cervical cancer studies evaluated obesity and BMI in relation to radical hysterectomy, minimally invasive surgery, laparotomy, postoperative morbidity, venous thromboembolism, infectious complications, lymphocele formation, and lower-limb lymphedema [6,18,24,33,42,50,52,67,73]. Several studies demonstrated that obesity or elevated BMI increased the risk of postoperative complications after cervical cancer surgery. Reported obesity-associated outcomes included deep vein thrombosis, infection, lower-extremity lymphedema, and short-term postoperative morbidity. However, the magnitude of association differed according to surgical approach, lymph node assessment, adjuvant treatment, and comorbidity burden [6,18,24,33,52,67,73].

In overweight or obese cervical cancer patients, minimally invasive approaches were generally associated with reduced blood loss, shorter hospitalization, and lower wound-related morbidity compared with open surgery, while overall complication rates remained relatively comparable across surgical techniques [6,18,24]. Lymphatic morbidity also represented a major postoperative issue in cervical cancer cohorts. BMI was frequently identified as a contributing factor for lower-limb lymphedema, particularly when combined with lymph node dissection, radiotherapy, chemotherapy, hypertension, infection, or reduced physical activity. These findings emphasize the multifactorial nature of lymphatic complications following cervical cancer treatment [43,50,67]. 

Several studies developed postoperative complication prediction models incorporating BMI alongside inflammatory markers, treatment-related variables, and surgical factors. These findings suggest that BMI contributes to perioperative risk stratification but should not be interpreted in isolation from other clinical variables [67,73]. Minimally invasive approaches may mitigate selected obesity-associated complications in carefully selected patients, although evidence remains heterogeneous [6,18,24,33,42,50,52,67,73].

Mixed gynecologic malignancy cohorts and other malignancies: Mixed gynecologic malignancy cohorts included studies evaluating gynecologic oncology laparotomy, minimally invasive surgery, pelvic exenteration, para-aortic lymphadenectomy, and perioperative morbidity across multiple gynecologic cancer types. These studies were retained when cancer-only populations or cancer-specific outcomes were clearly reported [5,31,32,34-36,44,54,55,64,68,70,72,75]. Across mixed gynecologic oncology cohorts, obesity and elevated BMI were consistently associated with increased perioperative risk, particularly wound complications, severe postoperative morbidity, venous thromboembolism, wound dehiscence, and major complications after extensive surgery. In high-complexity procedures such as pelvic exenteration or para-aortic lymphadenectomy, obesity interacted with additional variables including age, waist-to-hip ratio, surgical complexity, operative route, and comorbidity burden [5,31,32,34-36,44,54,55,72,76].

Studies evaluating para-aortic lymphadenectomy demonstrated that BMI and central adiposity measures, including waist-to-hip ratio, may independently predict surgical complications regardless of surgical approach. Robotic and extraperitoneal approaches appeared to reduce complication rates in selected analyses, although outcomes remained influenced by institutional experience and patient selection [64,68,70]. Lymphatic and thrombotic complications were also commonly represented in mixed gynecologic malignancy cohorts. Elevated BMI was associated with lower-extremity lymphedema after gynecologic cancer treatment and postoperative venous thromboembolism in uterine carcinosarcoma cohorts [36,44,76]. 

Evidence from uterine carcinosarcoma suggested that increased body habitus independently predicted postoperative venous thromboembolism and that thrombotic events were associated with poorer progression-free survival. Although derived from a specific high-risk malignancy, these findings reinforce the broader relationship between obesity, thrombosis, and adverse perioperative outcomes in gynecologic oncology surgery [76]. 

Overall Synthesis

**Figure 4:**
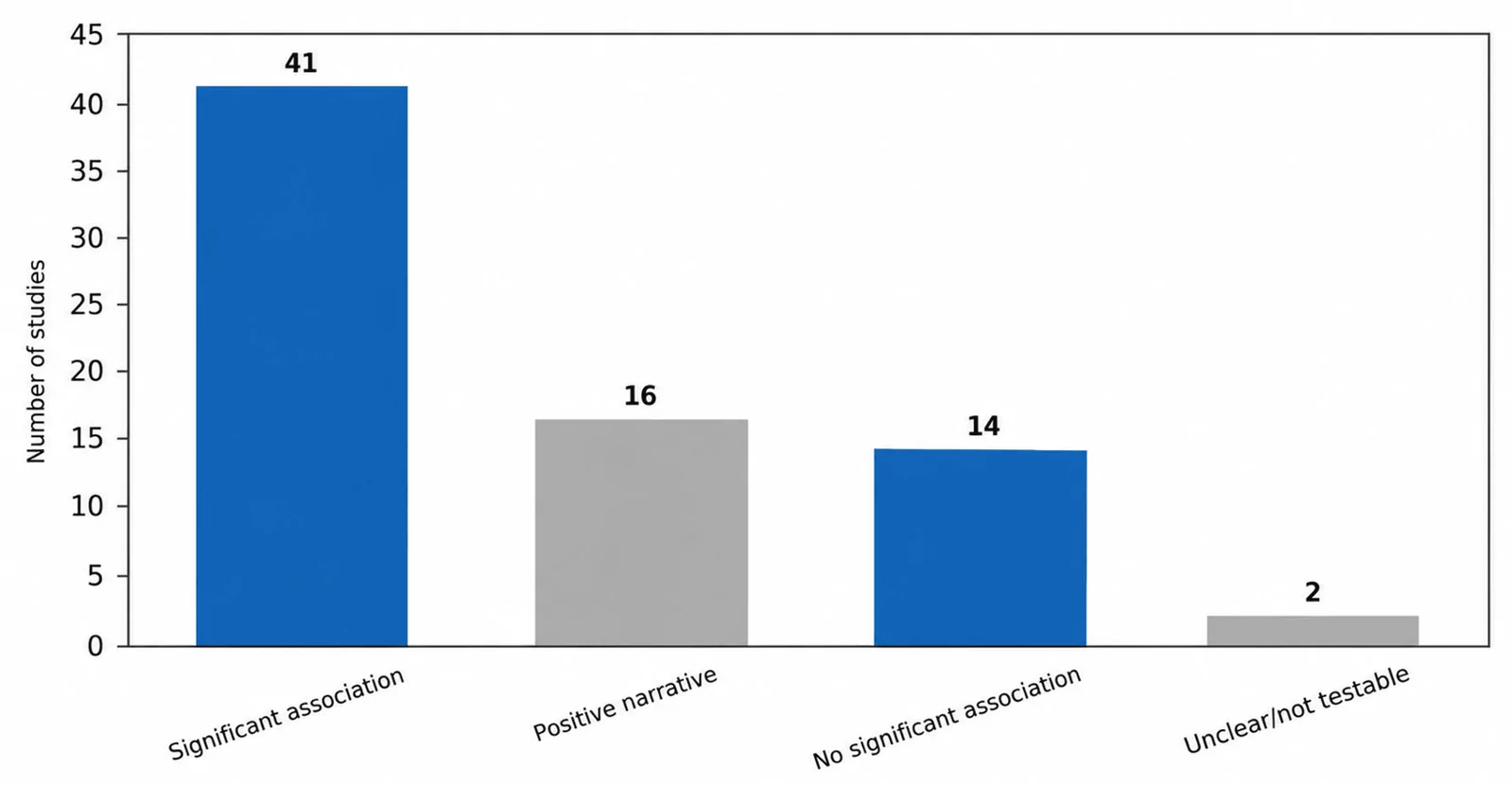
Direction and significance mapping of obesity/BMI/adiposity-related associations with perioperative morbidity across gynecologic oncology studies BMI: body mass index Bars represent a qualitative, vote-counting classification of reported study findings (not a pooled quantitative estimate); see Data Analysis for the categorization method

Across gynecologic oncology surgery, obesity, elevated BMI, and adiposity-related measures were most consistently associated with wound complications, surgical site infection, venous thromboembolism, lymphatic morbidity, operative difficulty, and prolonged recovery-related outcomes. Operative difficulty and recovery-related endpoints represented the most frequently reported morbidity category across studies, followed by wound complications and lymphatic morbidity (Figure 3). Direction-of-evidence mapping qualitatively demonstrated that most included studies reported significant associations between obesity-related measures and perioperative morbidity outcomes (Figure 4). Studies were categorized according to the reported direction of evidence into significant association, positive narrative association without formal statistical significance, no significant association, or unclear/non-testable evidence. This classification represented a qualitative synthesis of reported study findings rather than a pooled quantitative statistical analysis (Figure 4). The strength and consistency of associations varied according to cancer type, surgical approach, obesity definition, adiposity measure, and perioperative endpoint evaluated [1,5,23,31-45,76]. Summarized by exposure measure, BMI-based studies most consistently reported associations with wound complications and operative difficulty, whereas CT-defined visceral obesity, waist-to-hip ratio, and other body-composition measures were more consistently associated with pulmonary intolerance, postoperative fever, and delayed recovery, suggesting that different adiposity measures may capture partly distinct components of perioperative risk.

**Figure 5:**
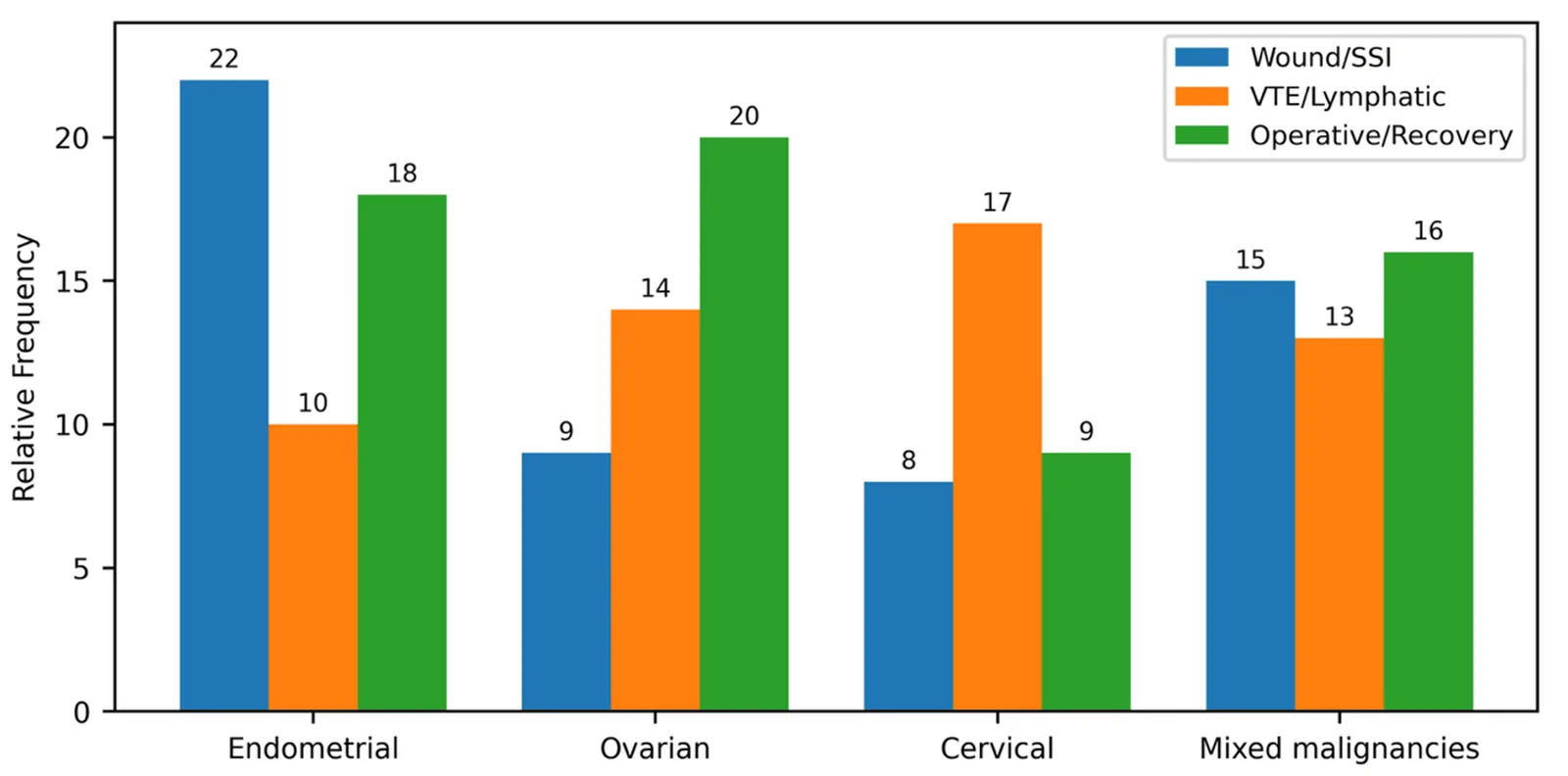
Comparison of obesity-associated perioperative morbidities across gynecologic malignancy groups

Comparison across gynecologic malignancy groups also demonstrated distinct morbidity patterns associated with obesity and adiposity measures (Figure 5). Endometrial cancer studies most frequently reported wound-related complications and operative/recovery difficulties, whereas cervical cancer studies more commonly emphasized venous thromboembolic and lymphatic morbidity. Ovarian, fallopian tube, and primary peritoneal cancer cohorts demonstrated substantial operative and recovery-related morbidity, reflecting the complexity of cytoreductive procedures and advanced-stage disease. Mixed gynecologic malignancy cohorts showed relatively balanced distributions across wound, lymphatic/thrombotic, and operative morbidity categories, highlighting the multifactorial perioperative impact of obesity across heterogeneous oncologic procedures.

The adverse effect of obesity appeared most clinically significant in open surgery and high-complexity procedures, including laparotomy, pelvic exenteration, cytoreductive surgery, and lymphadenectomy. In contrast, minimally invasive and robotic approaches were generally associated with acceptable perioperative outcomes in obese and morbidly obese patients and appeared to attenuate obesity-related morbidity in several studies [2-24]. Although BMI was the most frequently used obesity measure, several studies demonstrated that CT-defined visceral obesity, waist-to-hip ratio, perinephric fat, and body composition measures may provide additional perioperative risk stratification beyond BMI alone. 

Discussion

This systematic review evaluated the relationship between obesity, BMI, adiposity-related measures, and perioperative morbidity across gynecologic oncology procedures. Across the 73 included studies, obesity and elevated BMI were most consistently associated with wound complications, surgical site infection, venous thromboembolism, lymphatic morbidity, operative difficulty, and prolonged postoperative recovery-related outcomes [1,5,23,31-45,76]. Direction-of-evidence mapping further qualitatively demonstrated that most included studies reported significant associations between obesity-related measures and perioperative morbidity outcomes, whereas fewer studies demonstrated only positive narrative trends or no statistically significant association (Figure 4). Importantly, operative and recovery-related outcomes represented the most frequently reported morbidity category across studies, followed by wound complications and lymphatic morbidity. Nonetheless, the collective evidence suggests that obesity should be viewed not only as a technical surgical challenge but also as a broader indicator of perioperative vulnerability in gynecologic oncology patients.

The association between obesity and perioperative morbidity appeared to vary substantially according to cancer type, surgical complexity, and operative approach. Endometrial cancer represented the largest evidence subgroup, reflecting the strong epidemiologic relationship between obesity and endometrial carcinogenesis [2-4,7-17,19-23,63,77]. Across endometrial cancer cohorts, obesity was particularly associated with wound morbidity, surgical site infection, operative complexity, and prolonged recovery after open surgery [1,4,7,8,16,23,37,38,65]. However, minimally invasive and robotic-assisted surgery generally demonstrated favorable perioperative outcomes in obese and morbidly obese patients, including lower conversion rates, shorter hospitalization, reduced blood loss, and lower wound complication rates compared with laparotomy [2-4,7-17,19-23,63,77]. Although increasing BMI occasionally prolonged operative duration, this did not consistently translate into higher major postoperative morbidity, suggesting that minimally invasive approaches may attenuate part of the obesity-associated surgical risk [2,3,9-13,15,17,19-22].

Protective Role of Minimally Invasive and Robotic Surgery

The protective role of minimally invasive and robotic surgery represents one of the most clinically relevant findings of this review. Obese patients are particularly vulnerable to wound infection, dehiscence, pulmonary compromise, and delayed mobilization after large abdominal incisions [1,23,37,38,65]. By reducing incision size and tissue trauma, laparoscopic and robotic approaches may substantially decrease wound morbidity and shorten postoperative recovery [2-4,6-24]. Robotic surgery may additionally improve visualization, instrument articulation, and surgeon ergonomics during technically demanding procedures in high-BMI patients [2-4,6-24]. Nevertheless, the benefits of robotic surgery should be interpreted alongside operative cost, equipment availability, institutional experience, and anesthesia-related considerations, especially in morbidly obese patients undergoing prolonged procedures [7-13].

Limitations of BMI and the Role of Body Composition Measures

An additional important finding in our study was the limitation of BMI as the sole marker of obesity-related surgical risk. Several studies demonstrated that CT-defined visceral obesity, waist-to-hip ratio, perinephric fat thickness, and body composition analysis may provide more precise perioperative risk stratification than BMI alone [25-30]. These alternative adiposity-related measures were associated with pulmonary intolerance, increased operative complexity, delayed recovery, postoperative complications, and failed sentinel lymph node mapping in selected endometrial cancer cohorts [25-27]. Similarly, ovarian cancer studies demonstrated that visceral obesity and adverse body composition profiles were associated with postoperative fever, prolonged hospitalization, thrombotic complications, and severe postoperative morbidity after cytoreductive surgery [28-30]. These observations indicate that body composition and fat distribution may better reflect perioperative surgical risk than conventional BMI classification alone.

These comparisons should be interpreted cautiously, as most supporting studies were retrospective, single-center, and methodologically heterogeneous in how visceral obesity and body composition were measured and thresholded; larger prospective studies are needed before CT-derived adiposity measures can be considered superior to BMI in clinical practice, and routine clinical adoption is further limited by the additional imaging, software, and expertise required to derive these measures outside of research settings. The surgical approach itself (open versus minimally invasive or robotic) is a plausible confounder of the relationship between adiposity measures and perioperative outcomes, since more adverse body-composition profiles may have influenced the choice of operative approach; few included studies formally adjusted for this potential confounding. Clinically, identifying adverse body-composition profiles preoperatively may help target patients for enhanced preoperative optimization, closer perioperative monitoring, and more individualized counseling and surgical planning, particularly in ovarian cancer cohorts where visceral obesity was linked to postoperative fever and delayed recovery.

Differences Across Gynecologic Malignancies

The review additionally revealed essential differences in obesity-associated morbidity patterns across gynecologic malignancies (Figure 5). Endometrial cancer studies most frequently emphasized wound-related complications and operative/recovery difficulties, whereas cervical cancer studies more commonly reported venous thromboembolism, lymphatic complications, and lower-extremity lymphedema [6,18,24,33,42,50,52,67,73]. Ovarian, fallopian tube, and primary peritoneal cancer cohorts demonstrated substantial operative and recovery-related morbidity, likely reflecting the complexity of advanced cytoreductive procedures and the physiologic burden of advanced-stage disease [28-30,39-41,43,45,69]. Mixed gynecologic malignancy cohorts showed relatively balanced distributions across wound, thrombotic, lymphatic, and operative morbidity categories, highlighting the multifactorial perioperative impact of obesity across heterogeneous oncologic procedures [5,31,32,34-36,45,54,55,63,68,70,71,76]. These differences suggest that the relationship between obesity and perioperative morbidity is not uniform across gynecologic malignancies but is instead shaped by disease biology, surgical complexity, and typical operative approach for each cancer type. Consequently, pooling heterogeneous malignancies into a single mixed-cohort estimate, as required for several of the included analyses, may obscure cancer-specific risk patterns and limit the generalizability of pooled findings to any individual malignancy.

Among ovarian cancer cohorts, obesity-related morbidity appeared particularly influenced by disease burden, nutritional status, operative complexity, blood loss, and extent of cytoreduction [30,39-43,45,69]. Several studies identified elevated BMI as an independent predictor of thrombotic events, severe postoperative complications, and readmission after advanced ovarian cancer surgery [39,40,43,45]. Prominently, prediction-model studies frequently incorporated BMI alongside albumin level, inflammatory markers, surgical complexity, and disease stage, emphasizing that perioperative risk should be interpreted within a multifactorial clinical framework rather than by BMI alone [30,40,41,43]. Similarly, cervical cancer cohorts showed that obesity-related risk was modified by lymph node dissection, radiotherapy, infection, chemotherapy, and baseline comorbidity burden [42,50,52,67,73]. Another notable finding was the frequent association between obesity and lymphatic morbidity. Several studies demonstrated increased rates of lower-extremity lymphedema, lymphocele formation, and impaired lymphatic recovery in obese patients undergoing lymphadenectomy or nodal staging procedures [36,42,44,50,60,67,71]. Obesity and central adiposity were also associated with failed sentinel lymph node mapping in selected endometrial cancer studies [26,60,71]. These results are clinically significant because lymphatic complications may substantially impair long-term postoperative quality of life and functional recovery in gynecologic oncology patients.

Clinical Implications

The findings of this review have several important clinical implications. Obesity should not automatically exclude patients from surgical staging, cytoreductive surgery, minimally invasive surgery, or robotic-assisted approaches [2,3,5,14-17,19-22,29,43,64]. Instead, obesity should prompt individualized multidisciplinary perioperative planning involving gynecologic oncologists, anesthesiologists, perioperative teams, and the patient. Preoperative optimization of diabetes, cardiopulmonary comorbidities, nutritional status, and thromboembolic risk may be particularly important in obese patients undergoing complex procedures [1,23,32,34,35,37,38,43,65]. Enhanced recovery pathways, thromboprophylaxis, careful wound management, early mobilization, and individualized surgical planning may further reduce obesity-associated perioperative morbidity.

Limitations

Several limitations should be acknowledged when interpreting the findings of this review. Most included studies were observational, retrospective, single-center, or registry-based analyses, making the evidence susceptible to residual confounding, selection bias, institutional variability, and differences in perioperative management. Considerable heterogeneity was also present across studies regarding cancer type, surgical approach, obesity definition, BMI categorization, adiposity assessment, perioperative care pathways, outcome definitions, follow-up duration, and statistical adjustment methods. Consequently, the findings were more appropriately synthesized using a structured narrative analytical approach rather than a pooled quantitative meta-analysis. In addition, the qualitative direction-of-evidence mapping (Figure 4) reflected reported study trends rather than pooled statistical significance. Finally, although strict cancer-only eligibility criteria improved internal validity, some potentially relevant studies involving mixed benign or premalignant gynecologic populations were excluded because separate cancer-specific perioperative outcomes were not available.

Additionally, there are currently no standardized, widely accepted cutoffs for CT-defined visceral obesity, waist-to-hip ratio, or other body-composition measures, which limits direct comparability across studies and complicates translation of these measures into routine clinical risk-stratification tools. Second, differences in the specific imaging protocols, software, and anatomical landmarks used to derive adiposity measures across studies may have contributed to inconsistent findings independent of true biological differences. Third, because 37 of the 73 included studies (50.7%) were available only as conference abstracts, and abstract-only reporting was itself associated with moderate rather than low risk-of-bias ratings in several instances, the overall evidence base is weighted substantially toward less rigorously reported sources; findings drawn predominantly from full-text, peer-reviewed studies should be considered more robust than those relying heavily on abstract-only data, and this distinction should be borne in mind when interpreting the direction-of-evidence mapping. Finally, the qualitative vote-counting approach used in Figure 4 does not account for study sample size, effect magnitude, precision, or methodological quality and should not be interpreted as equivalent to a quantitative pooled estimate.

Future Research

Future research should aim to standardize how obesity-related surgical risk is measured and reported in gynecologic oncology. Future studies should report BMI categories consistently and should distinguish between overweight, obesity, morbid obesity, and class III obesity. In addition, studies should consider incorporating measures of central adiposity, visceral obesity, sarcopenic obesity, waist-to-hip ratio, and CT-based body composition to determine whether these measures improve risk prediction beyond BMI alone [25-30]. Prospective multicenter studies are needed to clarify the independent association between obesity and postoperative morbidity across different gynecologic malignancies and surgical procedures. Such studies should use standardized definitions for complications, wound morbidity, venous thromboembolism, lymphatic complications, conversion, readmission, and severe morbidity. They should also adjust for key confounders including age, comorbidities, performance status, cancer stage, surgical complexity, operative route, lymph node assessment, blood loss, and perioperative care pathways [5,30,37,38,40,43,64,68,69,72,73] as well as cost, access, surgeon experience, anesthesia safety, and oncologic outcomes [2-4,6-24]. 

## Conclusions

Obesity, elevated BMI, and adiposity-related body composition measures appear to be clinically important predictors of perioperative morbidity in gynecologic oncology surgery. Across multiple gynecologic malignancies, obesity was most consistently associated with wound complications, surgical site infection, venous thromboembolism, lymphatic morbidity, operative complexity, and prolonged postoperative recovery. However, the magnitude and consistency of these associations varied according to cancer type, surgical complexity, operative approach, and the specific obesity or adiposity measure evaluated. Minimally invasive and robotic-assisted approaches generally demonstrated acceptable perioperative outcomes in obese and morbidly obese patients and may reduce selected obesity-associated morbidities compared with open surgery, particularly in endometrial cancer. The findings also suggest that BMI alone may inadequately characterize perioperative risk, whereas CT-defined visceral obesity, waist-to-hip ratio, perinephric fat, and other body composition parameters may provide more refined risk stratification. Future prospective studies using standardized adiposity metrics and outcome definitions are needed to improve individualized perioperative risk assessment and surgical planning. As the current evidence base is predominantly observational and includes a substantial proportion of abstract-only reports, these conclusions should be regarded as hypothesis-generating rather than definitive, and are best applied to clinical practice and guideline development once confirmed by well-designed prospective studies using standardized adiposity and outcome definitions.

## Appendices

**Table 2:** Supplementary Table S1. Characteristics of the 73 included studies ACS-NSQIP, American College of Surgeons National Surgical Quality Improvement Program; AFR, albumin-to-fibrinogen ratio; BMI, body mass index; CT, computed tomography; DFS, disease-free survival; DVT, deep vein thrombosis; ER, emergency room; FIGO, International Federation of Gynecology and Obstetrics; GOG, Gynecologic Oncology Group; LOS, length of stay; NLR, neutrophil-to-lymphocyte ratio; NSQIP, National Surgical Quality Improvement Program; OS, overall survival; PALND, para-aortic lymph node dissection; SII, systemic immune-inflammation index; SLN, sentinel lymph node; SSI, surgical site infection; VO, visceral obesity; VTE, venous thromboembolism

No.	Study	Country	Study design	Cancer type	Surgical approach	Obesity/adiposity definition	N	Outcomes reported	Main findings
1	Bogani et al., 2021 [49]	Multi-institutional (country not specified)	Multi-institutional retrospective cohort study	Endometrial (high-risk)	Surgical staging for high-risk endometrial cancer, including pelvic and para-aortic lymphadenectomy and sentinel lymph node mapping	BMI analyzed as continuous predictor	279	90-day postoperative complications, severe complications (Clavien-Dindo grade ≥3), lymphatic-specific morbidity	Severe postoperative events occurred in 13 patients (4.6%). BMI and open abdominal surgery were independent predictors of surgery-related morbidity. Laparoscopic approach and sentinel node mapping were associated with lower surgery-related events
2	Nhokaew et al., 2015 [1]	Thailand	Retrospective cohort study	Endometrial	Laparotomy for surgical staging	BMI ≥30 kg/m²	357 (obese: 55)	Wound complications within 4 weeks: seroma, hematoma, wound separation, wound infection/SSI	Wound complication incidence was 7.84%. BMI ≥30, diabetes mellitus, and prior abdominal surgery were independent predictors of wound complications
3	Lechartier et al., 2023 [2]	Canada	Single-center retrospective cohort study	Endometrial	Robotic-assisted surgery for endometrial cancer	BMI ≥40 kg/m²; class III 40-49, class IV ≥50	185 (obese: 185; class III obesity n = 139, class IV obesity n = 46)	Intraoperative complications, postoperative complications by grade, grade 3-4 complications, conversion to laparotomy, estimated blood loss, length of hospital stay, readmission, recurrence	Robotic-assisted surgery for endometrial cancer was feasible and generally safe in morbidly and extremely morbidly obese patients. Class IV obesity was associated with a higher overall postoperative complication rate compared with class III obesity, but severe complications, conversion rate, blood loss, length of stay, readmission, and recurrence were low or similar between groups
4	Liu et al., 2023 [50]	China	Case-control study	Cervical	Surgical treatment for cervical cancer	BMI analyzed as a risk factor; specific BMI cutoff not reported in the abstract	759	Complications: postoperative lower-limb lymphedema	BMI was independently associated with postoperative lower limb lymphedema after cervical cancer surgery. This study supports BMI as a relevant risk factor for lymphatic morbidity after gynecologic cancer surgery
5	King et al., 2022 [3]	United States	Retrospective cohort study	Endometrial	Robotic-assisted surgical staging for endometrial cancer	Obesity defined as BMI >30 kg/m²; obesity classes categorized as class I, II, and III	391 (obese: 391)	Perioperative outcomes and postoperative complications	Postoperative complications appeared to increase with increasing BMI category among obese women undergoing robotic-assisted surgical staging for endometrial cancer. Although not statistically significant, the findings suggest a possible dose-response trend between higher obesity class and postoperative complications
6	Gambacorti-Passerini et al., 2019 [51]	Spain	Prospective observational study	Endometrial	Laparoscopic surgical staging for endometrial cancer	Patients classified as obese versus non-obese according to a standard BMI cutoff (BMI ≥30 kg/m²)	83 (obese: 46)	Operative time, number of lymph nodes removed, blood loss, length of hospital stay, intraoperative complications, postoperative complications, recurrence, time to recurrence, and survival	Obesity did not significantly worsen perioperative or oncologic outcomes among women with endometrial cancer undergoing laparoscopic surgical staging. Laparoscopic surgery appeared safe and effective in obese patients, with similar surgical complications, operative outcomes, recurrence, and survival compared with non-obese patients
7	Pyrzak et al., 2020 [31]	United States	Retrospective NSQIP targeted hysterectomy database study	Mixed gynecologic	Major abdominal or pelvic surgery for gynecologic malignancy	Obesity defined as BMI ≥30 kg/m²	20986	Complications; morbidity; readmission	Obesity was independently associated with increased risk of potentially avoidable 30-day readmission after surgery for gynecologic malignancy, but not with indicated readmission. This suggests that obese patients may be at higher risk for readmissions related to uncontrolled symptoms or minor complications after gynecologic oncology surgery
8	Abdelbadee et al., 2016 [25]	United States	Retrospective cohort study	Endometrial	Robotic hysterectomy and lymphadenectomy for endometrial cancer	BMI reported as continuous variable; mean BMI 34 kg/m²	59	Pulmonary complications, pulmonary intolerance during robotic surgery, measured by peak and plateau airway pressure, with average and maximum values assessed intraoperatively	CT-based morphometric adiposity measures, especially visceral fat volume at L2-L3, independently predicted pulmonary intolerance during robotic endometrial cancer surgery. Visceral adiposity may be more informative than BMI alone for predicting intraoperative pulmonary/anesthetic difficulty in obese patients undergoing robotic pelvic surgery
9	Wenzel et al., 2020 [51]	Netherlands	Nationwide historical cohort study/population-based registry study	Cervical	Radical hysterectomy with pelvic lymphadenectomy for early-stage cervical cancer	BMI analyzed as a continuous variable; mean BMI 26 ± 5 kg/m²	472	30-day intraoperative and postoperative surgical complications, overall surgical complications, urinary retention requiring catheterization, excessive perioperative blood loss (>1000 mL), and postoperative infection	Short-term surgical complications occurred in more than one-third of women after radical hysterectomy for early-stage cervical cancer. BMI was associated with overall complications in univariable analysis and independently predicted excessive perioperative blood loss, but BMI did not independently predict overall complications in the final multivariable model. Higher BMI was associated with a lower rate of urinary retention requiring catheterization
10	Hinshaw et al., 2016 [76]	United States	Retrospective cohort study	Endometrial	Robotic-assisted hysterectomy versus open abdominal hysterectomy for endometrial cancer	Class II obesity defined as BMI >35 kg/m²; class III obesity defined as BMI >40 kg/m²	136 (obese: 136)	Postoperative complications, length of hospital stay, blood loss, operative time, lymph node dissection rate, and pelvic and para-aortic lymph node yield	Robotic hysterectomy appeared safe and effective in morbidly obese women with endometrial cancer. Compared with open abdominal hysterectomy, robotic surgery was associated with reduced perioperative morbidity, including fewer postoperative complications, shorter hospitalization, and lower blood loss, without compromising lymph node assessment, adjuvant treatment use, or oncologic outcomes
11	Durdağ et al., 2023 [4]	Turkey	Retrospective cohort study	Endometrial (high-risk)	Complete surgical staging for endometrioid endometrial cancer, including hysterectomy, bilateral salpingo-oophorectomy, and pelvic lymphadenectomy	BMI >30 kg/m² subgroup; mean BMI overall 35.85 ± 8.30 kg/m²	278 (obese: 202)	Blood loss and length of stay: operation duration, hemoglobin drop as a blood-loss indicator, first flatus time, oral alimentation time, drain removal time, length of hospital stay, and intraoperative and postoperative complications	Laparoscopic staging for endometrioid endometrial cancer was associated with better perioperative recovery than laparotomy, including shorter time to flatus and shorter hospital stay, including among patients with BMI >30 kg/m². In obese patients, laparoscopy had longer operative time but similar hemoglobin drop and shorter hospitalization. Intraoperative complication rates were not significantly different between laparoscopy and laparotomy
12	Kılıç et al., 2021 [32]	Turkey	Retrospective cohort study	Mixed gynecologic	Elective gynecologic oncology surgery performed through a xiphoidopubic (vertical midline) incision	Obesity defined as BMI >30 kg/m² in the methods; BMI also analyzed as a continuous variable	198	Postoperative evisceration/abdominal wound dehiscence, wound complications, recurrence and survival in the advanced ovarian/fallopian tube/peritoneal cancer subgroup, and delay to adjuvant chemotherapy	High BMI/obesity was an independent predictor of postoperative evisceration after gynecologic oncology surgery. Smoking, preoperative hypoalbuminemia, and high ASA score were also independent predictors. Evisceration represents a specific severe wound complication and, although it delayed chemotherapy initiation, it was not associated with recurrence or survival in the advanced ovarian/fallopian tube/peritoneal cancer subgroup
13	Levin et al., 2024 [5]	United States	Retrospective NSQIP database study	Mixed gynecologic	Pelvic exenteration for gynecologic malignancy (anterior, posterior, or total)	BMI analyzed as continuous predictor; mean BMI 27.6 ± 8.1 kg/m²	794	Complications and wound outcomes: 30-day postoperative complications, major complications, minor complications, organ/space SSI, superficial SSI, urinary tract infection, and blood transfusion	Major postoperative complications occurred in about one-third of women undergoing pelvic exenteration for gynecologic malignancy, and overall complications were very common. Higher BMI was independently associated with increased odds of major postoperative complications. Diabetes, low albumin, and elevated creatinine were also independent predictors. The study supports BMI as a clinically relevant predictor of major complications after pelvic exenteration
14	Cheng et al., 2024 [33]	China	Retrospective study	Cervical	Surgical treatment for cervical cancer; open surgery and laparoscopic/robotic-assisted surgery	BMI analyzed as a risk factor/continuous variable. Mean BMI analyzed as a risk factor/continuous variable	282	Postoperative lower extremity deep venous thrombosis within 1 month after surgery; pulmonary embolism among DVT patients; risk factors for postoperative DVT	BMI/obesity was independently associated with postoperative lower extremity DVT after cervical cancer surgery. Advanced age, elevated preoperative D-dimer, higher triglyceride level, and open surgery were also independent risk factors. This study supports obesity/BMI as a relevant predictor of thromboembolic morbidity after gynecologic cancer surgery
15	Peng et al., 2017 [54]	Canada	Retrospective cohort study	Mixed gynecologic	Laparoscopic gynecologic oncology procedures performed by gynecologic oncology surgeons	Non-obese BMI <30 kg/m²; obese BMI 30–39.9 kg/m²; morbidly obese BMI ≥40 kg/m²	497 (obese: 209; obese BMI 30-39.9 n = 162, morbidly obese BMI ≥40 n = 47)	Operative time, estimated blood loss, intraoperative transfusion, postoperative transfusion, conversion to laparotomy, intraoperative complications, major intraoperative complications, postoperative complications, severe postoperative complications, readmission, and emergency room visits	Laparoscopic gynecologic oncology surgery appeared feasible and safe in obese and morbidly obese patients. Higher BMI was associated with longer operative time, higher estimated blood loss, and more intraoperative transfusions, but conversion to laparotomy, intraoperative complications, severe postoperative complications, readmission, and ER visits did not differ significantly between BMI groups
16	Lei et al., 2017 [6]	China	Retrospective cohort study	Cervical	Laparoscopic radical hysterectomy for cervical cancer; Piver type II laparoscopic radical hysterectomy for cervical cancer (Piver type II or III)	Obesity defined as BMI ≥25 kg/m²; non-obese defined as BMI <25 kg/m²	243 (obese: 64)	Operative time, intraoperative blood loss, conversion to open surgery, return of bowel movement, postoperative hospital stay, intraoperative complications, 30-day postoperative complications, recurrence, and 5-year survival	In cervical cancer patients undergoing laparoscopic radical hysterectomy, obesity defined as BMI ≥25 kg/m² was associated with increased operative difficulty, including longer operative time, greater blood loss, and higher conversion to open surgery. However, obesity did not significantly increase intraoperative complications, 30-day postoperative complications, postoperative stay, recurrence, or 5-year survival
17	Milman et al. 2024 [55]	United States	Retrospective NSQIP database cohort study	Mixed gynecologic	Elective minimally invasive hysterectomy for gynecologic oncologic indications	BMI analyzed by categories: <30, 30-34.9, 35-39.9, 40-49.9, and ≥50 kg/m²	32823 (obese: not reported as a combined total; BMI categories reported individually as 30-34.9, 35-39.9, and 40-49.9 kg/m²)	Morbidity and same-day discharge: same-day discharge, postoperative day 1 discharge, 30-day composite morbidity, readmission, unplanned reoperation, UTI, SSI, wound disruption, and pneumonia	Same-day discharge after minimally invasive hysterectomy for gynecologic oncologic indications increased substantially over time and appeared safe, with no increase in 30-day composite morbidity. However, high BMI, especially BMI ≥40 kg/m², was a barrier to same-day discharge and was independently associated with higher 30-day composite morbidity. The study supports BMI as an important predictor of both discharge timing and postoperative morbidity
18	Palaiologos et al., 2025 [56]	United Kingdom	Retrospective cohort study	Endometrial	Robotic hysterectomy for endometrial cancer	Class III obesity defined as BMI ≥40 kg/m²	236 (obese: 236; all patients had class III obesity)	Robotic surgery completion rate, conversion to laparotomy, perioperative events, inpatient length of stay, infection, and laparotomy rate over time	Robotic hysterectomy was feasible and safe in most patients with class III obesity and endometrial cancer. The study showed high robotic completion rates, low conversion to laparotomy, short inpatient stay, and low infection rate. Outcomes appeared to improve over time, with a significant reduction in laparotomy rates across the 13-year experience
19	Uccella et al., 2015 [57]	Italy	Multicenter retrospective case-control study with multivariable and propensity-score matched analysis	Endometrial	Surgical treatment for endometrial cancer by laparoscopic or open abdominal surgery	Obesity defined by BMI categories (obese: BMI ≥30 kg/m²); obese patients n = 391	1266 (obese: 391)	Overall postoperative complications, incidence and severity of postoperative complications, wound complications, venous thromboembolic events, blood transfusion, and postoperative hospital stay and lymphadenectomy performance	Obesity was associated with higher perioperative morbidity after endometrial cancer surgery, particularly wound complications and venous thromboembolic events. However, laparoscopy retained significant perioperative advantages over open surgery even among obese patients, including fewer complications, shorter hospital stay, less transfusion, and better likelihood of lymphadenectomy. Morbid obesity was associated with the greatest perioperative risk
20	Cybulska et al., 2017 [58]	-Not reported	Retrospective cohort study	Endometrial	Minimally invasive staging surgery for endometrial cancer, robotically assisted or standard laparoscopic	BMI analyzed by category; BMI ≥40 kg/m² used as a high-BMI threshold	760 (obese: 101 patients with BMI ≥40 kg/m²)	Trocar site hernia formation, hernia location, and time to hernia diagnosis	Minimally invasive staging surgery for endometrial cancer had a low overall trocar site hernia rate. Robotic and standard laparoscopic approaches had similar hernia rates. Higher BMI, specifically BMI ≥40 kg/m², was significantly associated with postoperative trocar site hernia formation
21	Januszek et al., 2019 [59]	Poland	Prospective observational study	Endometrial	Traditional open abdominal surgical treatment for endometrial cancer, including hysterectomy, bilateral salpingo-oophorectomy, and lymphadenectomy	Obesity defined as BMI ≥30 kg/m². Additional adiposity: obesity defined as BMI ≥30 kg/m²; additional adiposity assessed by waist circumference	70 (obese: 37)	Duration of operation, duration of hospitalization, perioperative hemoglobin loss, procedure-related complications, wound infection/impaired wound healing, bladder injury, and gastrointestinal injury	Obesity was associated with worse in-hospital perioperative outcomes after traditional open surgery for endometrial cancer, including longer operative time, longer hospitalization, and greater hemoglobin loss. Waist circumference appeared to be the strongest adiposity predictor and may be useful for operative risk stratification. The study did not demonstrate a statistically significant association between BMI and wound infection.
22	Boitano et al., 2021 [34]	United States	Retrospective cohort study/pre-post enhanced recovery protocol implementation study	Mixed gynecologic	Non-emergent laparotomy for pathology-proven gynecologic malignancy	Obesity defined as BMI ≥30 kg/m²; morbid obesity defined as BMI ≥40 kg/m²	363 (obese: 275 patients with BMI ≥30 kg/m²; control group obese n = 85, enhanced recovery protocol group obese n = 169; morbidly obese BMI ≥40: total n = 64, control n = 21, enhanced recovery protocol n = 43)	Overall postoperative complications, Clavien-Dindo grade I-II and III-IV complications, length of stay, 30-day readmission, ileus, pulmonary complications, and surgical site infection	Implementation of an enhanced recovery protocol was associated with lower overall complication rates and shorter hospital stay among gynecologic oncology patients undergoing non-emergent laparotomy, without increasing 30-day readmissions. The benefit was particularly notable among obese and morbidly obese patients, with a large reduction in complications in patients with BMI> 40. Reductions were mainly driven by the implementation of an enhanced recovery protocol, which was associated with lower overall complication rates and shorter hospital stay among gynecologic oncology patients undergoing nonemergent laparotomy, without increasing 30-day readmissions. The benefit was particularly notable among obese and morbidly obese patients, with a large reduction in complications in patients with BMI> 40. Reductions were mainly driven by fewer wound and pulmonary complications
23	Straubhar et al., 2024 [35]	United States	Retrospective cohort study/pre-post quality improvement study	Mixed gynecologic	Incisional hernia within 1 year, postoperative infection, surgical site. Vertical midline laparotomy with small-bite versus large-bite fascial closure technique for gynecologic oncology surgery	BMI analyzed as a risk factor. Obesity was included in BMI analysis as a risk factor; obesity was included as a covariate in the multivariable model	255	Incisional/trocar hernia: Incisional or trocar-site hernia within 1 year, postoperative wound infection, and surgical site complications	Small bite fascial closure reduced incisional hernia rates after vertical midline laparotomy in gynecologic oncology patients. Obesity was a strong independent predictor of incisional hernia, and the benefit of small bite closure appeared greatest among patients with higher BMI. Chemotherapy was also an independent risk factor for hernia formation
24	Sofer et al., 2020 [7]	Israel	Retrospective observational cohort study	Endometrial	Robotic surgery versus open surgery for low-grade endometrial cancer, total hysterectomy with bilateral salpingo-oophorectomy	Obesity defined as BMI ≥30 kg/m²	138 (obese: 138; all patients had BMI ≥30 kg/m²)	Operating room time, skin-to-skin procedure time, length of hospital stay, postoperative complications within 30 days, Clavien-Dindo grade ≥II complications, wound infection, and respiratory complications	Among obese women with low-grade endometrial cancer, robotic surgery was associated with fewer postoperative complications, fewer Clavien-Dindo grade ≥II complications, shorter hospital stay, faster recovery, and better quality-of-life measures compared with open surgery. Robotic surgery required longer operative/theater time, but survival was not compromised. This study supports robotic surgery as the preferred approach for obese women with low-grade endometrial cancer
25	Deura et al., 2019 [8]	Japan	Retrospective cohort study	Endometrial	Surgical staging for presumed low-risk endometrial cancer. Laparoscopic surgical staging for presumed low-risk endometrial cancer; laparoscopic versus laparotomy approaches compared	BMI categorized as <25, 25–<30, and ≥30 kg/m²; obesity BMI categorized as <25, 25–<30, and ≥30 kg/m²; obesity defined as BMI ≥30 kg/m²	120 (obese: 19 patients with BMI ≥30 kg/m²; laparoscopy n = 4 and laparotomy n = 15)	Operative time, estimated blood loss, number of pelvic lymph nodes removed, postoperative hospital stay, intraoperative complications, postoperative complications, and wound complications	Laparoscopic staging surgery for presumed low-risk endometrial cancer was feasible and safe compared with laparotomy. Laparoscopy was associated with less blood loss, shorter hospital stay, and more pelvic lymph nodes retrieved, without increasing intraoperative or postoperative complications. Although laparoscopic surgery took longer overall, this difference disappeared among obese patients with BMI ≥30 kg/m²
26	Leitao et al., 2019 [60]	United States	Retrospective cohort study with patient-reported outcome measures	Endometrial	Primary surgery for newly diagnosed endometrial cancer, including hysterectomy and lymph node assessment	BMI analyzed as a continuous variable; median BMI was similar across comparison groups	599	Patient-reported lower-extremity lymphedema, quality of life, effect of sentinel lymph node mapping versus lymphadenectomy, and effect of EBRT and BMI on lymphedema prevalence	In endometrial cancer patients undergoing primary surgery, higher BMI was associated with increased prevalence of patient-reported lower-extremity lymphedema. Sentinel lymph node mapping was associated with lower lymphedema prevalence compared with comprehensive lymph node dissection, even after adjusting for BMI and EBRT. This study supports BMI as a risk factor for lymphatic morbidity after endometrial cancer treatment
27	Mendivil et al., 2015 [9]	United States	Retrospective cohort study	Endometrial	Hysterectomy, bilateral salpingo-oophorectomy, and pelvic lymphadenectomy	Morbid obesity defined as BMI ≥40 kg/m²; median BMI comparable across surgical groups	53 (obese: 53; all patients had morbid obesity with BMI ≥40 kg/m²)	Operative time, estimated blood loss, blood transfusion, intraoperative complications, conversion to laparotomy, postoperative complications, pelvic lymph node yield, and length of hospital stay	In morbidly obese patients with endometrial cancer, minimally invasive surgery was associated with lower estimated blood loss and shorter hospital stay compared with open surgery, although operative time was longer. Postoperative complications were numerically more frequent in the open surgery group, but the study was small. Both robotic-assisted and conventional laparoscopic approaches appeared feasible in this morbidly obese population
28	Chan et al., 2015 [10]	United States	Retrospective Nationwide Inpatient Sample database study	Endometrial	Surgical treatment for endometrial cancer using open, laparoscopic, or robotic approaches	Morbid obesity defined as BMI ≥40 kg/m²	1087 (obese: 1087; all patients had morbid obesity with BMI ≥40 kg/m²)	Intraoperative and postoperative complications, blood transfusion, mechanical ventilation, urinary tract injury, gastrointestinal injury, wound debridement, infection, and venous thromboembolism	In morbidly obese endometrial cancer patients, minimally invasive surgery, either robotic or laparoscopic, was associated with fewer perioperative complications and shorter hospitalization compared with open surgery. Robotic surgery had complication rates comparable to laparoscopy but higher total charges
29	Carbajal-Mamani et al., 2021 [61]	-Not reported	Retrospective cohort study	Endometrial	Robotic-assisted hysterectomy/robotic-assisted surgery for newly diagnosed endometrial cancer	Obesity/high-BMI cohort defined as BMI ≥35 kg/m²	132 (obese: 132; all patients had BMI ≥35 kg/m²)	30-day postoperative venous thromboembolism, in-hospital VTE, perioperative complications, estimated blood loss, readmission, use of pharmacologic thromboprophylaxis, and use of pneumatic compression devices	Among obese patients with BMI ≥35 kg/m² undergoing robotic-assisted surgery for newly diagnosed endometrial cancer, 30-day postoperative VTE risk was low. Extended 4-week prophylaxis was not associated with a statistically significant reduction in VTE compared with no extended prophylaxis, although only one VTE event occurred. Perioperative complications and readmissions were similar between groups
30	Ayres et al., 2025 [62]	Australia	Single-arm prospective observational pilot study	Endometrial	Laparoscopic surgery for low-risk endometrial cancer after 4-6 weeks of a very low-calorie diet	Severe obesity defined as BMI ≥35 kg/m²; mean baseline BMI reported at enrollment	25 analyzed of 28 enrolled (obese: 25 analyzed; all patients had severe obesity with BMI ≥35 kg/m²)	Safety and feasibility of the very low-calorie diet, weight loss, BMI reduction, waist/hip circumference, blood pressure, biochemical changes, estimated blood loss, perioperative complications, and length of hospital stay	A 4-6 week pre-operative Optifast very low-calorie diet was feasible, acceptable, and safe in severely obese patients with clinically low-risk endometrial cancer. The intervention achieved significant short-term reductions in weight, BMI, waist and hip circumference, and diastolic blood pressure. All patients proceeded to laparoscopic surgery without major adverse events, conversion to laparotomy, or serious adverse events related to the diet intervention
31	Salman et al., 2025 [26]	-Not reported	Retrospective cohort study	Endometrial	Surgical staging for endometrial cancer with sentinel lymph node mapping	BMI analyzed as continuous predictor; additional adiposity measured by perinephric fat thickness on CT	297	Sentinel lymph node mapping success/failure, bilateral failed mapping, intraoperative complications, postoperative complications, and survival	Higher BMI and increased average perinephric fat were associated with higher risk of bilateral failed sentinel lymph node mapping
32	Ozdemir et al., 2022 [63]	Turkey	Retrospective single-center cohort study	Endometrial	Endometrial cancer surgery by laparotomy, conventional laparoscopy, or robotic surgery	BMI classified according to WHO categories: normal weight, overweight, obese, and morbidly obese	142 (obese: 45)	Surgical procedure, estimated blood loss, surgery time, ASA/preoperative health status, duration of hospital stay, number of total dissected pelvic lymph nodes, and metastatic and nonmetastatic lymph node status	Obesity was associated with longer operative time in patients undergoing surgery for endometrial cancer, but was not significantly associated with higher blood loss, longer hospital stay, surgical approach, perioperative complications, or in-hospital mortality. The authors concluded that both minimally invasive surgery and conventional laparotomy could be performed safely in obese patients with endometrial cancer.
33	Bebia et al., 2021 [64]	Spain	Post hoc analysis of a prospective randomized open-label trial	Mixed (endometrial/ovarian)	Minimally invasive para-aortic lymphadenectomy for surgical staging of endometrial or ovarian cancer	BMI analyzed as continuous predictor; waist-to-hip ratio also analyzed	203	Composite PALND-related surgical complications, blood loss ≥500 mL, intraoperative PALND-related complications, severe postoperative complications (Clavien-Dindo grade ≥IIIA), and inability to complete lymphadenectomy	Higher BMI and higher waist-to-hip ratio were independent predictors of complications during minimally invasive para-aortic lymphadenectomy for endometrial or ovarian cancer staging. Robot-assisted extraperitoneal PALND was associated with fewer surgical complications without compromising lymph node retrieval, operative time, or length of stay
34	Gracia et al., 2020 [11]	Spain	Comparative retrospective cohort study	Endometrial	Robotically assisted laparoscopy versus standard laparoscopy for endometrial cancer	BMI groups included BMI <25, BMI ≤30, and BMI ≥30 kg/m²	234 (obese: not reported as a total; BMI ≥30 subgroup reported but exact total obese N not reported in the abstract)	Hospital stay, estimated blood loss, conversion to laparotomy, perioperative outcomes, and surgical complications	In endometrial cancer surgery, robotically assisted laparoscopy showed perioperative advantages compared with standard laparoscopy, especially among obese patients. In BMI ≥30 patients, robotic surgery was associated with lower blood loss and significantly lower conversion to laparotomy. These findings support robotic surgery as a useful approach for obese women with endometrial cancer
35	Ramzan et al., 2015 [65]	United States	Retrospective cohort study using a prospectively collected institutional database	Endometrial	Hysterectomy-based surgical staging for endometrial cancer by panniculectomy-combined laparotomy, standard laparotomy, or minimally invasive surgery	BMI classified as <30 kg/m², 30-39.9 kg/m², 40-49.9 kg/m², and ≥50 kg/m²	551 (obese: 356 patients with BMI ≥30 kg/m²; morbidly obese BMI 40-49.9 n = 105; superobese BMI ≥50 n = 50)	Intraoperative major complications, postoperative major complications within 30 days, surgical-site wound complications, VTE, ileus, sepsis/bacteremia, pelvic abscess, and unplanned reoperation	Panniculectomy-combined surgical staging for endometrial cancer was feasible in highly obese/superobese patients but was associated with increased postoperative major complications compared with laparotomy and MIS. MIS had the lowest complication and wound complication rates. Surgical mortality was not increased with panniculectomy. The authors suggest panniculectomy may be considered selectively. Panniculectomy-combined surgical staging for endometrial cancer was feasible in highly obese/superobese patients but was associated with increased postoperative major complications compared with laparotomy and MIS. MIS had the lowest complication and wound complication rates. Surgical mortality was not increased with panniculectomy. The authors suggest panniculectomy may be considered selectively, in carefully chosen highly obese patients
36	Iavazzo et al., 2021 [66]	Greece	Retrospective single-center case series/cohort study	Mixed (endometrial/ovarian)	Gynecologic cancer surgery with concurrent panniculectomy	Morbidly obese cohort; median BMI 43.79 kg/m²	38 (obese: 38; all patients were obese/morbidly obese)	Operative time, blood loss, intraoperative complications, postoperative complications, wound infection, wound dehiscence, renal failure, and mortality	Concurrent panniculectomy with gynecologic cancer surgery was reported as feasible in selected morbidly obese patients and may improve surgical exposure. However, wound morbidity occurred, including wound infection in 5 patients and one death after wound infection, wound dehiscence, and renal failure
37	Kadoch et al., 2024 [12]	Canada	Retrospective cohort study	Endometrial	Robotic surgery for endometrial cancer, including total hysterectomy, bilateral salpingo-oophorectomy, and pelvic/para-aortic lymphadenectomy or sentinel lymph node mapping	Non-obese BMI <30 kg/m², obese BMI 30.0-39.9 kg/m², morbidly obese BMI ≥40 kg/m²	1329 (obese: 753; BMI 30.0-39.9 n = 449 and BMI ≥40 n = 304; additional BMI ≥50 subgroup n = 69)	Operating room time, set-up time, skin-to-skin/procedure time, postoperative time, estimated blood loss, conversion to laparotomy, PACU stay, length of hospitalization, intraoperative complications, and 30-day emergency room presentations	Robotic surgery for endometrial cancer showed comparable perioperative safety across BMI groups. Higher BMI was associated with longer overall OR time, mainly from increased set-up time, and slightly higher EBL, but there were no significant differences in conversion to laparotomy, hospital stay, PACU stay, intraoperative complications, or 30-day ER presentations. Robotic surgery remained feasible and safe across all BMI categories
38	Mendivil et al., 2016 [36]	-Not reported	Retrospective cohort study/5-year review	Mixed gynecologic	Surgical management of endometrial or cervical cancer with lymphadenectomy	BMI >35 kg/m² analyzed as a risk factor for lower-extremity lymphedema	165	Postoperative lower-extremity lymphedema, pelvic lymph node positivity, para-aortic lymph node positivity, and risk factors for lymphedema	The overall incidence of postoperative lower-extremity lymphedema after lymphadenectomy for endometrial and cervical cancer was low. However, BMI >35 kg/m² and multiple comorbidities were significant risk factors for lymphedema. The study supports high BMI as a risk factor for lymphatic morbidity after gynecologic cancer surgery
39	Mizuno et al., 2025 [13]	Japan	Retrospective cohort study	Endometrial	Robot-assisted hysterectomy with bilateral salpingo-oophorectomy and pelvic lymphadenectomy	Obesity defined as BMI ≥30 kg/m²; BMI <30 group n = 117, BMI ≥30 group n = 88	197 (obese: 88)	Operative time, console time, open/closure time, blood loss, postoperative length of hospital stay, intraoperative complications, postoperative complications, pelvic infection, and urinary tract infection	In patients undergoing robotic surgery for stage IA endometrial cancer, BMI ≥30 did not significantly worsen operative time, blood loss, hospital stay, perioperative complication rates, or cancer-specific survival. However, obesity was associated with higher comorbidity burden, higher treatment costs, and more lifestyle-related diseases during follow-up. BMI >35 was associated with higher postoperative in-patients undergoing robotic surgery for stage IA endometrial cancer; BMI ≥30 did not significantly worsen operative time, blood loss, hospital stay, perioperative complication rates, or cancer-specific survival. However, obesity was associated with higher comorbidity burden, higher treatment costs, and more lifestyle-related diseases during follow-up. BMI >35 was associated with higher postoperative cost
40	Åkesson et al., 2021 [37]	Sweden	Population-based retrospective cohort study using the Swedish Quality Register for Gynecologic Cancer	Endometrial	Primary surgical staging for endometrial cancer, including hysterectomy and lymph node assessment	BMI categorized as <30 vs ≥30 kg/m²BMI categorized as <30 versus ≥30 kg/m²	556 identified; complications data available for 549 (obese: 232 patients with BMI ≥30 kg/m²)	Surgical complications within 30 days classified by Clavien-Dindo, clinically significant complications (CD grade II–V), any complication (CD grade I–V), estimated blood loss, and blood transfusion	In endometrial cancer surgery, obesity, open surgery, and lymphadenectomy were independently associated with clinically significant surgical complications. Robotic/minimally invasive surgery was associated with fewer complications. Complications were associated with worse short-term overall survival. The study supports minimizing surgical morbidity, especially in obese patients, and strengthens the case for minimally invasive surgery
41	Pace et al., 2026 [27]	Italy	Retrospective cohort study	Endometrial	Minimally invasive surgery for apparent early-stage endometrial cancer	BMI-based obesity defined as BMI ≥30 kg/m²; abdominal obesity defined by waist-to-hip ratio >0.85	151 (obese: 66 patients with BMI ≥30 kg/m²; abdominal obesity by waist-to-hip ratio >0.85 in 127 patients)	Operative time, sentinel lymph node mapping success/failure, conversion to laparotomy, Clavien-Dindo grade ≥2 postoperative complications, readmission, reoperation, and length of stay	CT-measured waist-to-hip ratio, an abdominal adiposity measure, was independently associated with longer operative time in minimally invasive surgery for early-stage endometrial cancer, whereas BMI category was not. Neither WHR >0.85 nor BMI ≥30 was associated with increased postoperative complications or sentinel node mapping failure. The study supports the use of body composition/adiposity measures such as waist-to-hip ratio as complementary predictors to BMI
42	Cheng et al., 2016 [14]	China	Retrospective cohort study comparing laparoscopic with open surgery	Endometrial	Hysterectomy with pelvic lymphadenectomy; laparoscopic surgery versus open surgery	Morbid obesity defined as BMI >40 kg/m²	120 (obese: 120; all patients were morbidly obese with BMI >40 kg/m²)	Operative time, estimated blood loss, pelvic lymph node yield, para-aortic node yield, length of hospital stay, intraoperative complications, postoperative complications, and blood transfusion	In morbidly obese patients with early-stage endometrial cancer, laparoscopic surgery was associated with significantly lower intraoperative and postoperative complications, lower blood loss, and shorter hospital stay compared with open surgery, with similar operative time and lymph node yield. The authors concluded that laparoscopic surgery is a safe and rational therapeutic strategy for morbidly obese patients with early-stage endometrial cancer
43	Li et al., 2026 [67]	China	Cross-sectional study with development and validation of a nomogram prediction model	Cervical	Postoperative cervical cancer patients; lymph node dissection performed as part of primary surgical treatment	BMI analyzed as an independent predictor; exact BMI cutoff not specified in the abstract	Not reported in abstract	Postoperative lower limb lymphedema, independent predictors of LLL, nomogram model performance, AUC, calibration, and decision curve analysis	BMI was an independent predictor of lower limb lymphedema after cervical cancer surgery. The developed nomogram showed good predictive performance for identifying postoperative cervical cancer patients at risk of LLL
44	Cunningham et al., 2015 [15]	-Not reported	Retrospective cohort study	Endometrial	Planned robotic surgery for endometrial carcinoma, with lymph node dissection as indicated	Non-obese BMI 19-29 kg/m², obese BMI 30-39 kg/m², morbidly obese BMI 40-71 kg/m²	298 (obese: 211; obese BMI 30-39 n = 110 and morbidly obese BMI 40-71 n = 101)	Conversion to laparotomy, operative room time, surgical time, complications, lymphadenectomy performance, number of lymph nodes retrieved, and previous abdominal surgery as a risk factor	Increasing BMI was not associated with higher conversion to laparotomy or complication rates among patients undergoing robotic surgery for endometrial carcinoma. Morbid obesity increased operating room time but did not compromise surgical time, lymphadenectomy performance, or lymph node yield
45	Mahdi et al., 2014 [38]	United States	Retrospective NSQIP database cohort study	Endometrial	Surgery for endometrial cancer, including laparoscopy and laparotomy	Normal weight BMI 18 to <30 kg/m², obese BMI 30 to <40. Normal weight BMI 18 to <30 kg/m², obese BMI 30 to <40 kg/m², morbidly obese BMI ≥40 kg/m²	3947 (obese: Approximately 2447; obese BMI 30 to <40 = 38%, morbidly obese BMI ≥40 = 24%)3947 (obese: approximately 2447; obese BMI 30 to <40 = 38%, morbidly obese BMI ≥40 = 24%)	30-day morbidity, 30-day mortality, any postoperative complication, surgical complications, infectious complications, laparoscopic surgery rate, and laparotomy subgroup complications and mortality by BMI subgroup	Morbidly obese endometrial cancer patients had more preoperative comorbidities and higher crude 30-day postoperative morbidity, especially surgical and infectious complications, and were less likely to undergo minimally invasive surgery. However, obesity was not an independent predictor of 30-day morbidity or mortality after adjustment. Laparoscopy appeared to mitigate BMI-related differences in complication rates
46	Nie et al., 2024 [28]	-Not reported	Retrospective cohort study	Ovarian/tubal/peritoneal	Cytoreductive surgery for advanced epithelial ovarian cancer	CT-based visceral obesity measured by visceral fat area	130 (obese: 70 patients with visceral obesity)	Short-term postoperative complications, postoperative fever, length of hospital stay, time from surgery to adjuvant chemotherapy, visceral fat area, subcutaneous fat area, and total fat area	CT-defined visceral obesity was associated with higher short-term postoperative complications in patients with advanced ovarian cancer undergoing cytoreductive surgery. Visceral obesity was independently associated with postoperative complications and was linked to postoperative fever and longer hospital stay
47	Urunsak et al., 2020 [16]	Turkey	Retrospective cohort study	Endometrial	Total hysterectomy and bilateral salpingo-oophorectomy with or without pelvic lymphadenectomy	Morbid obesity defined as BMI ≥40 kg/m² according to WHO criteria	146 (obese: 146; all patients were morbidly obese with BMI ≥40 kg/m²)	Operation time, intraoperative complications, postoperative complications, postoperative hospital stay, lymphadenectomy, lymph node involvement, adjuvant treatment, disease-free survival, and overall survival	In morbidly obese patients with endometrial cancer, laparoscopy was associated with fewer postoperative complications and shorter hospital stay compared with laparotomy, without significant differences in five-year DFS or OS. Omission of lymphadenectomy was associated with shorter operative time, fewer intraoperative and postoperative complications, and shorter hospital stay, without significant differences in oncologic outcomes
48	Díaz-Feijoo et al., 2020 [68]	Spain	Prospective randomized multicenter study	Mixed (endometrial/ovarian)	Minimally invasive para-aortic staging lymphadenectomy using a laparoscopic extraperitoneal or transperitoneal approach	BMI analyzed as continuous predictor; waist-to-hip ratio also analyzed	203	Composite surgical complication outcome, bleeding during para-aortic lymphadenectomy ≥500 mL, intraoperative PALND-related complication, and severe postoperative complication (Clavien-Dindo grade ≥IIIA)	In early-stage endometrial and ovarian cancer patients undergoing minimally invasive para-aortic staging lymphadenectomy, extraperitoneal and transperitoneal approaches had similar surgical complication rates and oncologic outcomes. Higher BMI and higher waist-to-hip ratio were independent predictors of surgical complications
49	Asanoma et al., 2022 [17]	Japan	Retrospective institutional cohort study	Endometrial	Robotic-assisted surgery for stage IA endometrial cancer	Non-obese BMI <30 kg/m², obese BMI ≥30 to <35 kg/m², morbidly obese BMI ≥35 kg/m²	62 (obese: 23; obese BMI ≥30 to <35 n = 13 and morbidly obese BMI ≥35 n = 10)	Conversion to laparotomy, total operation time, setting time (including trocar installation and docking), console time, wound closure time, blood loss, perioperative complications, and 30-day readmission	Robotic-assisted surgery appeared safe for obese and morbidly obese patients with stage IA low-risk endometrial cancer. Higher BMI was not associated with increased blood loss or perioperative complications, and conversion was uncommon
50	Mahdi et al., 2016 [69]	United States	Retrospective ACS-NSQIP database cohort study	Ovarian/tubal/peritoneal	Surgery for ovarian cancer, including at least salpingo-oophorectomy and cytoreductive procedures as indicated	Non-obese BMI <30 kg/m², obese BMI 30 to <40 kg/m², morbidly obese BMI ≥40 kg/m²	2061 (obese: 725; obese BMI 30 to <40 n = 560 and morbidly obese BMI ≥40 n = 165)	30-day morbidity, 30-day mortality, any complication, surgical complications, nonsurgical complications, surgical site infection, renal complications, pulmonary complications, and septic complications	In ovarian cancer surgery, obesity and morbid obesity were not independent predictors of overall 30-day morbidity or mortality after adjustment for confounders. However, morbidly obese patients had higher surgical site infection rates, especially superficial SSI. The study suggests that short-term surgical morbidity may not explain poorer long-term outcomes reported in obese ovarian cancer patients. In ovarian cancer surgery, obesity and morbid obesity were not independent predictors of overall 30-day morbidity or mortality after adjustment for confounders. However, morbidly obese patients had higher surgical site infection rates, especially superficial SSI. The study suggests that short-term surgical morbidity may not explain poorer long-term outcomes reported in obese ovarian cancer patients
51	Chen et al., 2024 [18]	China	Retrospective cohort study	Cervical	Radical hysterectomy with pelvic/para-aortic lymph node dissection by open, laparoscopic, or robotic approach	Asian BMI criteria: overweight BMI ≥24.0 kg/m² and obesity BMI ≥28.0 kg/m²	382 (obese: 382 overweight or obese patients according to Asian BMI criteria)	Operating time, estimated blood loss, blood transfusion, return of bowel movement, postoperative hospital stay, lymph node yield, abdominal organ injury, and surgery-related nerve injury	Among overweight or obese patients with stage IB–IIA cervical cancer, robotic radical hysterectomy was associated with shorter operative time, lower blood loss, shorter bowel recovery time, and shorter postoperative stay compared with open and laparoscopic approaches. Open surgery had higher wound complication and infection rates. Robotic surgery had higher urinary retention and pelvic lymphocele rates
52	Reyes Claret et al., 2020 [70]	-Not reported	Retrospective observational cohort study	Mixed gynecologic	Transperitoneal laparoscopic para-aortic lymphadenectomy for para-aortic staging in gynecologic malignancy	BMI <30 kg/m² vs BMI ≥30 kg/m²BMI <30 kg/m² versus BMI ≥30 kg/m²	146 (obese: 40 patients with BMI ≥30 kg/m²)	Lymph node count, intraoperative complications, postoperative complications, feasibility rate, operating time, and hospital stay	BMI≥30 did not significantly reduce feasibility or lymph node yield and was not associated with higher intraoperative or postoperative complications or longer hospital stay. However, obesity was associated with significantly longer operating time. The authors concluded that obesity by BMI alone should not be considered a limiting factor for transperitoneal laparoscopic para-aortic lymphadenectomy
53	Raventós-Tato et al., 2019 [19]	Spain	Retrospective cohort study	Endometrial	Surgical staging for endometrial cancer by laparotomy, conventional laparoscopy, or robotic surgery	High-BMI cohort defined as BMI ≥35 kg/m²	138 (obese: 138; all patients had BMI ≥35 kg/m²)	Operative bleeding, hospital admission length, early postoperative complications, lymphadenectomy rate, number of lymph nodes removed, recurrence-free survival, and overall survival	In women with endometrial cancer and BMI ≥35 kg/m², minimally invasive surgical staging was associated with lower blood loss and shorter hospital stay compared with laparotomy. Robotic surgery had fewer early postoperative complications. Oncologic outcomes, including recurrence-free and overall survival, were not compromised
54	Backes et al., 2015 [20]	United States	Retrospective single-institution cohort study	Endometrial	Robotic-assisted hysterectomy with or without complete surgical staging	Obesity defined as BMI ≥30 kg/m²; BMI categories analyzed up to BMI >50 kg/m²	543 (obese: 380 patients with BMI ≥30 kg/m²)	Postoperative complications, severe postoperative complications, ICU admission, reintubation, reoperation, perioperative death, readmission, fever, ileus, and bleeding requiring transfusion	In patients undergoing robotic hysterectomy for endometrial cancer, increasing BMI was not significantly associated with higher overall or severe postoperative complications. There was a non-significant trend toward more complications in patients with BMI >50 and in patients with ≥3 comorbidities. Cardiac and renal comorbidities were associated with the highest observed complication rates
55	Kim et al., 2022 [39]	-Not reported	Retrospective single-institution cohort study	Ovarian/tubal/peritoneal	Debulking surgery for ovarian cancer	BMI analyzed as an independent risk factor; low-risk group defined by age <57 years and BMI <21 kg/m²	799	Postoperative thrombotic events within 6 months, pulmonary embolism, deep vein thrombosis, other thrombotic events, prophylaxis strategy, risk factors for thrombosis, and overall survival	Postoperative thrombotic event incidence after ovarian cancer debulking surgery was low. BMI, age, and operative duration were independent predictors of thrombotic events. A young and lean subgroup, age <57 and BMI <21, had very low thrombotic risk, suggesting de-escalated prophylaxis may be considered in selected low-risk patients
56	Matanes et al., 2021 [71]	Not reported	Retrospective cohort study	Endometrial	Surgical staging for endometrial cancer using sentinel lymph node sampling	Obesity/high-BMI cohort defined as BMI ≥35 kg/m²; median BMI reported at enrollment	223 (obese: 223; all patients had BMI ≥35 kg/m²)	Operative time, estimated blood loss, lymph node assessment, intraoperative adverse events, postoperative adverse events, progression-free survival, overall survival, and disease-specific survival	In obese patients with endometrial cancer, omitting lymphadenectomy and using SLN sampling was associated with shorter operative time and lower estimated blood loss without compromising 2-year survival outcomes. This supports SLN sampling as a morbidity-reducing staging approach in obese patients
57	Inci et al., 2020 [72]	Germany	Prospective clinical exploratory cohort study (RISC-GYN trial)	Mixed gynecologic	Elective surgery for gynecological cancer, including hysterectomy/adnexectomy and, where indicated, bowel resection	BMI categorized as underweight <20, normal 20-25, overweight 25-30, and obese >30 kg/m²	226 (obese: 60 patients with BMI >30 kg/m²; overweight n=30 with BMI >25 to <30 kg/m²)	Severe postoperative complications within 30 days, Clavien-Dindo grade ≥IIIb complications, postoperative mortality, wound infection/dehiscence, pulmonary embolism, sepsis, and anastomotic leak	In gynecological cancer surgery, overweight/obesity was a strong independent predictor of severe postoperative complications. Nutritional/body composition status measured by bioelectrical impedance phase angle, ECOG performance status, physical functioning, norepinephrine requirement, and colon resection also predicted severe complications. This study supports BMI and body composition/performance in gynecological cancer surgery; overweight/obesity was a strong independent predictor of severe postoperative complications. Nutritional/body composition status measured by bioelectrical impedance phase angle, ECOG performance status, physical functioning, norepinephrine requirement, and colon resection also predicted severe complications. This study supports BMI and body composition/performance-status measures as complementary preoperative risk-stratification tools
58	Kawai et al., 2021 [21]	France	Retrospective cohort study	Endometrial	Robotic surgery for endometrial cancer	Obesity defined as BMI ≥30 kg/m²	175 (obese: 42)	5-year overall survival, 5-year recurrence-free survival, laparotomy conversion, type of surgery, pelvic lymphadenectomy, adjuvant treatment, and postoperative morbidity/complications	Obese patients with endometrial cancer undergoing robotic surgery had similar conversion rates, postoperative complication rates, and oncologic outcomes compared with non-obese patients. Robotic surgery appears safe in obese patients with endometrial cancer, with low morbidity and no apparent survival disadvantage
59	Kumar et al. 2015 [40]	United States	Retrospective cohort study/risk prediction model study	Ovarian/tubal/peritoneal	Primary debulking surgery with curative intent for advanced epithelial ovarian cancer	BMI analyzed categorically; BMI ≥40 kg/m² and BMI <25 kg/m² analyzed as risk categories	620 (obese: NR; BMI ≥40 kg/m² group analyzed)620 (obese: not reported as a total; BMI ≥40 kg/m² group analyzed)	Severe postoperative complications, Accordion grade ≥3 complications, 90-day mortality, surgical complexity, ASA score, preoperative albumin, FIGO stage, and nomogram/risk prediction model performance	BMI was an important predictor in risk models for severe complications and 90-day mortality after primary debulking surgery for advanced epithelial ovarian cancer. Morbid obesity BMI ≥40 was associated with a higher risk of severe complications and 90-day mortality, while low BMI <25 also appeared associated with increased risk. The model supports using BMI alongside age, ASA score, albumin, stage, and surgical complexity to predict severe postoperative morbidity and mortality
60	Inci et al., 2021 [73]	Germany	Prospective cohort study; ovarian cancer subgroup analysis of the RISC-GYN trial	Ovarian/tubal/peritoneal	Maximal cytoreductive surgery for primary ovarian cancer	BMI categories: underweight <20 kg/m², normal 20-25 kg/m², overweight 25-30 kg/m², obese >30 kg/m²	106 (obese: 20 patients with BMI >30 kg/m²; overweight n = 28 with BMI 25-30 kg/m²)	Severe postoperative complications within 30 days, Clavien-Dindo grade ≥IIIb complications, postoperative mortality, wound dehiscence, anastomotic insufficiency, and thromboembolic events	In primary ovarian cancer, patients undergoing maximal cytoreductive surgery; overweight/obesity defined as BMI >25 kg/m² was a strong independent predictor of severe postoperative complications. ECOG performance status >1, high intraoperative norepinephrine requirement, and high fresh frozen plasma transfusion were also independent predictors. The study supports BMI and ECOG as practical preoperative risk-stratification tools
61	Xing et al., 2024 [41]	China	Retrospective single-center cohort study/nomogram prediction model study	Cervical	Selective laparoscopic hysterectomy for cervical cancer under general anesthesia	BMI ≥24.0 kg/m² used as an overweight/obesity threshold according to Asian BMI criteria	301 (obese: not reported as a total; BMI ≥24 group n = 64)	Postoperative infectious complications within 1 month, surgical site infection, respiratory infection, urinary tract infection, and nomogram prediction model incorporating age, BMI, diabetes, SII, AFR, and NLR	In cervical cancer patients undergoing laparoscopic hysterectomy, BMI ≥24 was an independent risk factor for postoperative infectious complications. The study developed a nomogram incorporating age, BMI, diabetes, SII, AFR, and NLR with strong predictive performance for postoperative infection risk
62	de Jong et al., 2024 [42]	Netherlands	Retrospective single-center cohort study	Mixed gynecologic	Robot-assisted laparoscopic pelvic lymphadenectomy as part of primary surgical treatment for cervical or endometrial cancer	BMI analyzed as a continuous variable in kg/m²; no obesity-specific threshold applied	387	Symptomatic lymphocele, grade 2–3 lymphocele, readmission, drainage, intravenous antibiotics, deep vein thrombosis related to lymphocele, and risk factors for symptomatic lymphocele	Symptomatic lymphocele occurred in nearly 10% of patients after robot-assisted pelvic lymphadenectomy for cervical or endometrial cancer. BMI was not a significant predictor in the combined cohort or endometrial cancer subgroup, but higher BMI independently predicted symptomatic lymphocele in cervical cancer patients. Smoking was the main overall risk factor
63	Joshi et al., 2024 [22]	India	Retrospective single-center cohort study	Endometrial	Robotic-assisted complete staging surgery for endometrial cancer, including hysterectomy, bilateral salpingo-oophorectomy, and lymphadenectomy or sentinel node mapping	Low-BMI group BMI <30 kg/m²; high-BMI/obese group BMI ≥30 kg/m²	99 (obese: 60 patients with BMI ≥30 kg/m²; morbidly obese subgroup n=13 with BMI ≥40 kg/m²)	Operative time, estimated blood loss, length of hospital stay, early postoperative complications, delayed postoperative complications, Clavien-Dindo classification, lymph node yield, recurrence, disease-free survival, and overall survival	In this Indian tertiary-care robotic surgery cohort for endometrial cancer, BMI ≥30 was not associated with worse perioperative or oncologic outcomes. Operative time, blood loss, LOS, early and delayed complications, lymph node yield, recurrence, DFS, and OS were similar between BMI groups. The morbidly obese subgroup also had favorable perioperative outcomes with no reported complications
64	Simpson et al., 2020 [23]	Canada	Retrospective population-based cohort study with matched-cohort analysis	Endometrial	Primary surgical management with hysterectomy for endometrioid endometrial cancer	Class III obesity defined as BMI ≥40 kg/m², identified via administrative/health-records data	12112 (obese: 2697 patients with class III obesity; matched cohort included 2320 class III obesity patients matched 1:1 to patients without class III obesity)	30-day perioperative complications, composite complication rate, wound infection/disruption, and complications by surgical approach (open versus minimally invasive surgery)	In a population-based cohort of women undergoing hysterectomy for endometrioid endometrial cancer, class III obesity was associated with higher overall 30-day perioperative complications, mainly due to increased wound infection/disruption. However, when minimally invasive surgery was used, women with class III obesity had similar complication risk to women without class III obesity
65	Heus et al., 2021 [29]	Netherlands	Retrospective cohort study	Ovarian/tubal/peritoneal	Primary or interval cytoreductive/debulking surgery for advanced ovarian cancer	CT-based visceral obesity defined as visceral fat area above a predefined threshold on CT imaging	298 (obese: 122 patients with visceral obesity)	Overall postoperative complications within 30 days, Clavien-Dindo complications, thrombotic events, wound infection, ileus, pneumonia, urinary tract infection, abscess, sepsis, and reoperation	In patients with advanced ovarian cancer undergoing cytoreductive surgery, CT-defined visceral obesity was strongly associated with postoperative complications and independently predicted complications after adjustment. BMI and skeletal muscle mass were not independent predictors. The study supports CT-based visceral adiposity as a more clinically useful risk marker than BMI alone
66	Bacalbasa et al., 2020 [74]	Romania	Retrospective cohort study	Ovarian/tubal/peritoneal	Cytoreductive/debulking surgery with curative intent for advanced-stage ovarian cancer	BMI analyzed as a preoperative risk factor; median BMI reported at baseline	80	Serious postoperative complications requiring reoperation within 30 days, postoperative mortality, intra-abdominal hemorrhage, pancreatic abscess/leak, urinary leak, and digestive leak	In advanced epithelial ovarian cancer patients undergoing cytoreductive surgery, higher BMI/obesity, older age, poor nutritional status measured by low albumin, and comorbidity burden were associated with serious postoperative complications requiring reoperation. The study supports considering BMI and nutritional status during preoperative risk assessment
67	Castro et al., 2018 [43]	Brazil	Retrospective cohort study	Ovarian/tubal/peritoneal	Primary debulking surgery or interval debulking surgery for advanced ovarian cancer	Obesity defined as BMI >30 kg/m²	83 (obese: 13 patients with BMI >30 kg/m²)	30-day postoperative complications, minor complications, major complications, adjuvant chemotherapy delay, 30-day reoperation, 30-day rehospitalization, fever, and surgical wound infection	In advanced ovarian cancer patients undergoing primary or interval debulking surgery, BMI >30 did not predict 30-day postoperative complications, but it independently predicted delay in adjuvant chemotherapy. Surgical morbidity was mainly associated with comorbidities and higher estimated blood loss, while chemotherapy delay was associated with obesity, hypertension, reoperation, and postoperative complications
68	Güner et al., 2024 [75]	-Not reported	Retrospective cohort study	Mixed gynecologic	Midline laparotomy / vertical incision surgical procedure for gynecologic malignancy	Overweight/obesity defined as BMI ≥25 kg/m²	72 (obese: 72 patients with BMI ≥25 kg/m²)	Postoperative wound complications within 8 weeks, seroma, hematoma, superficial dehiscence, fascial dehiscence, wound infection, and hospital stay	In overweight/obese gynecologic cancer patients undergoing midline laparotomy, suture closure showed a trend toward fewer wound complications compared with staples and was associated with shorter hospital stay. The difference in overall wound complications did not reach statistical significance
69	Hayes et al., 2017 [44]	-Not reported	Prospective longitudinal cohort study	Mixed gynecologic	Surgery for gynecological cancer, with varying extent of lymph node dissection	BMI analyzed as a continuous/increasing risk factor; no specific cutoff applied	488	Lower-limb lymphedema prevalence, lower-limb lymphedema incidence, self-reported lymphedema, objectively measured lymphedema by bioimpedance spectroscopy, persistent lymphedema, and lymphedema-related quality of life	Lower-limb lymphedema was common before and after gynecological cancer surgery. Increasing BMI was identified as a potential risk factor for lymphedema, along with extent of lymph node dissection, adjuvant therapy, physical inactivity, vulvar/vaginal cancer, and baseline lymphedema
70	Duska et al., 2015 [45]	United States	Secondary analysis of GOG 0218/NRG Oncology-Gynecologic Oncology Group trial	Mixed gynecologic	Upfront cytoreductive surgery followed by front-line chemotherapy on GOG protocol 0218	BMI analyzed as continuous/risk factor; mean/median BMI not specified in the abstract	1873	Readmission within 30 days of previous admission or within 40 days of cytoreductive surgery, postoperative readmission, multiple readmissions, and time from surgery to chemotherapy initiation	In patients with ovarian, fallopian tube, or primary peritoneal carcinoma receiving upfront surgery and chemotherapy in GOG 0218, higher BMI independently increased odds of readmission. Most readmissions were postoperative, suggesting the early post-surgical period is an important target for intervention
71	Su et al., 2025 [30]	China	Retrospective cohort study	Ovarian/tubal/peritoneal	Radical ovarian cancer surgery, including comprehensive staged surgery	CT-defined visceral obesity using visceral fat area measurement	309 (obese: visceral obesity group: 201 total; younger age ≤65 years VO n = 140; older age >65 years VO n = 61)	Postoperative complications, Clavien-Dindo grade ≥2 complications, ICU admission, intestinal anastomotic fistula, shock, organ failure, and postoperative lower-extremity deep vein thrombosis	CT-defined visceral obesity was associated with postoperative complications in ovarian cancer, particularly in patients aged ≤65 years. VO was a better risk marker than BMI in this cohort. In older patients, FIGO stage rather than visceral obesity predicted postoperative complications
72	Xu et al., 2025 [24]	China	Retrospective cohort study	Cervical	Laparoscopic radical hysterectomy or open radical hysterectomy for early-stage cervical cancer	Overweight/obesity defined as BMI ≥24 kg/m², likely using Asian BMI criteria	112 (obese: 112 overweight/obese patients with BMI ≥24 kg/m²)	Intraoperative blood loss, hospital stay, postoperative complications, wound fat liquefaction, overall survival, and disease-free survival	In overweight/obese women with early-stage cervical cancer, laparoscopic radical hysterectomy was associated with lower blood loss, shorter hospital stay, and fewer wound fat liquefaction events compared with open radical hysterectomy, with similar overall complications and oncologic outcomes
73	Matsuo et al., 2017 [76]	Multiple countries	Multicenter retrospective cohort study	Uterine carcinosarcoma	Primary surgical treatment for stage I–IV uterine carcinosarcoma	Body habitus/BMI analyzed continuously in kg/m²; obesity defined per study-specific criteria	906	Postoperative venous thromboembolism, cumulative VTE incidence, VTE risk factors, progression-free survival, and survival impact of VTE	In women with uterine carcinosarcoma undergoing primary surgery, higher BMI/body habitus independently increased postoperative VTE risk. Obese non-Asian women with large tumors had a particularly high VTE risk. VTE was also associated with worse progression-free survival, suggesting it may reflect aggressive disease and poorer patient condition

**Table 3:** Supplementary File S2. Studies excluded after full-text/abstract eligibility reassessment, with reasons EIN: endometrial intraepithelial neoplasia

No.	Study (author, year)	Reported cancer population	Reason for exclusion
1	Harrison et al., 2020 [78]	Mixed gynecologic cancer: suspected or confirmed	Mixed suspected gynecologic cancer cohort with benign cases; cancer-only outcomes not separable
2	Fabregó et al., 2024 [79]	Ovarian cancer and borderline ovarian tumors	Mixed ovarian cancer and borderline ovarian tumors; cancer-only outcomes not separable
3	Moulton et al., 2017 [80]	Mixed gynecologic cancer	Mixed gynecologic oncology cohort including adnexal masses and endometrial hyperplasia/cancer; cancer-only outcomes not separable
4	Lennox et al., 2020 [81]	Mixed gynecologic cancer	Cancer-only eligibility unclear; main outcome same-day discharge rather than postoperative complication
5	DeMari et al., 2022 [82]	Gynecologic oncology abdominal hysterectomy	Cancer-only population unclear; no separate cancer-specific outcomes reported in abstract
6	Litt et al., 2025 [83]	Mixed gynecologic cancer: mostly gynecologic	Mixed cancer/premalignant/benign cohort; cancer-only outcomes not separable; main outcome same-day discharge
7	Tewari et al., 2022 [84]	Mixed gynecologic cancer	Gynecologic oncology indications cohort; cancer-only population unclear and cancer-specific outcomes not separable
8	Damiani et al. 2019 [85]	Mixed endometrial cancer and endometrial hype	Mixed endometrial cancer and endometrial hyperplasia cohort; cancer-only outcomes not separable
9	Thomas et al., 2016 [86]	Suspected gynecologic malignancy; confirmed m	Mixed suspected malignancy cohort; only 66% malignant and cancer-only outcomes not separable.
10	Lynam et al., 2016 [87]	Suspected or confirmed gynecologic malignancy	Mixed suspected/confirmed gynecologic malignancy cohort; not all patients had malignancy and cancer-only outcomes not separable.
11	Ditto et al., 2025 [88]	Mixed gynecologic disease cohort; cancer-only	Mixed cancer, premalignant, borderline, and benign cohort; cancer-only outcomes not separable.
12	Giannini et al., 2024 [89]	Mixed endometrial hyperplasia and endometrial	Mixed endometrial hyperplasia and endometrial cancer cohort; cancer-only outcomes not separable.
13	Fennimore et al., 2023 [90]	Mixed premalignant EIN and clinical stage 1 e	Mixed EIN and endometrial cancer cohort; cancer-only outcomes not separable; main outcome not postoperative complication
14	Suidan et al., 2017 [91]	Mixed endometrial carcinoma and endometrial h	Mixed endometrial carcinoma and endometrial hyperplasia cohort; cancer-only outcomes not separable
15	Burdette et al., 2024 [92]	Cancer type: Mixed endometrial cancer and end	Mixed endometrial cancer and EIN cohort; cancer-only outcomes not separable; main outcome same-day discharge.
